# Diffusion tensor imaging, intra-operative neurophysiological monitoring and small craniotomy: Results in a consecutive series of 103 gliomas

**DOI:** 10.3389/fonc.2022.897147

**Published:** 2022-09-13

**Authors:** Giorgio Carrabba, Giorgio Fiore, Andrea Di Cristofori, Cristina Bana, Linda Borellini, Barbara Zarino, Giorgio Conte, Fabio Triulzi, Alessandra Rocca, Carlo Giussani, Manuela Caroli, Marco Locatelli, Giulio Bertani

**Affiliations:** ^1^ Neurosugery, Azienda Socio Sanitaria Territoriale Monza - Ospedale San Gerardo di Monza, Monza, Italy; ^2^ Dipartimento di Medicina e Chirurgia, Università degli Studi di Milano-Bicocca, Milan, Italy; ^3^ Neurosurgery, Fondazione IRCCS Ca’ Granda Ospedale Maggiore Policlinico, Università degli Studi di Milano, Milan, Italy; ^4^ Neurophysiopathology, Fondazione IRCCS Ca’ Granda Ospedale Maggiore Policlinico, Università degli Studi di Milano, Milano, Italy; ^5^ Neuroradiology, Fondazione IRCCS Ca’ Granda Ospedale Maggiore Policlinico, University of Milano, Milano, Italy; ^6^ Department of Medical-Surgical Physiopathology and Transplantation, University of Milan, Milan, Italy

**Keywords:** glioma, neurosurgery, neurophysiological monitoring (IOM), DTI - diffusion tensor imaging, mini craniotomy, glioblastoma multiforme (GBM), low grade glioma (LGG)

## Abstract

Diffusion tensor imaging (DTI) allows visualization of the main white matter tracts while intraoperative neurophysiological monitoring (IONM) represents the gold standard for surgical resection of gliomas. In recent years, the use of small craniotomies has gained popularity thanks to neuronavigation and to the low morbidity rates associated with shorter surgical procedures. The aim of this study was to review a series of patients operated for glioma using DTI, IONM, and tumor-targeted craniotomies. The retrospective analysis included patients with supratentorial glioma who met the following inclusion criteria: preoperative DTI, intraoperative IONM, tumor-targeted craniotomy, pre- and postoperative MRI, and complete clinical charts. The DTI was performed on a 3T scanner. The IONM included electroencephalography (EEG), transcranial (TC) and/or cortical motor-evoked potentials (MEP), electrocorticography (ECoG), and direct electrical stimulation (DES). Outcomes included postoperative neurological deficits, volumetric extent of resection (EOR), and overall survival (OS). One hundred and three patients (61 men, 42 women; mean age 54 ± 14 years) were included and presented the following WHO histologies: 65 grade IV, 19 grade III, and 19 grade II gliomas. After 3 months, only three patients had new neurological deficits. The median postoperative volume was 0cc (IQR 3). The median OS for grade IV gliomas was 15 months, while for low-grade gliomas it was not reached. In our experience, a small craniotomy and a tumor resection supported by IONM and DTI permitted to achieve satisfactory results in terms of neurological outcomes, EOR, and OS for glioma patients.

## Introduction

Diffusion tensor imaging (DTI) is an advanced neuroimaging technique that allows the 2D and 3D reconstruction of important white matter tracts such as cortico-spinal tract (CST), superior longitudinal fascicles (SLFs), and inferior frontal-occipital (IFO) fascicles (1–4). Preservation of white matter tracts is crucial for patients’ neurological integrity and, probably, even more than the preservation of certain cortical areas. Several publications on DTI confirm its reliability and role in planning neurosurgical resection of brain tumors (1–3, 5, 6). Knowing in advance the tridimensional architecture of the main fiber bundles surrounding a brain tumor can help in choosing the best approach to the tumor and preserve essential white matter tracts (2, 7, 8). As a consequence, DTI can contribute to decreasing the postoperative neurological deficit, reducing surgical invasiveness, and helping in tailoring the most appropriate craniotomy (9–11).

When considering brain tumor resections, intraoperative neurophysiological monitoring (IONM) and brain mapping techniques represent the gold standard for surgical management of primary CNS neoplasms (12, 13). These neurophysiological techniques allow to identify and preserve important cortical and subcortical functional sites while monitoring fundamental neurological functions (e.g., MEP and SSEP - (1, 5, 14–16)). Brain mapping usually requires the exposure of a large area of the brain cortex to identify the different eloquent sites (e.g., primary motor strip, speech arrest sites). Eloquent areas are determined by positive responses to direct electrical stimulation (DES) of the motor areas or by the interference of speech for language (17, 18). Thus, relatively large classic frontotemporal craniotomies are generally performed to obtain enough space for tumor removal and positive mapping. Nevertheless, in recent years the use of small craniotomies targeted on the tumor has gained popularity thanks to neuro-navigation systems and the low morbidity rates associated with shorter surgical procedures, reduced anesthetic drug administration, and consequently shorter patients’ hospitalization (7, 8, 19, 20). Only recently, some authors have advocated the utility of negative mapping without necessarily obtaining positive sites to have safer tumor resections (15, 21). Few studies are available in the literature dealing with the intriguing technical topic of the combination of mini-craniotomy (or tumor-targeted craniotomy or again small craniotomy) and the use of IONM. The two techniques are somehow considered mutually exclusive (how can you have a positive response site when you perform a tumor-targeted craniotomy)? or compatible in a small number of cases (e.g., tumor nearby of eloquent sites).

In this work, we present the experience of the authors in a consecutive series of 103 patients affected by astrocytoma grades I to IV operated combining DTI, IOM, and minimally invasive craniotomy. Technical aspects will be presented, together with neurological outcomes, surgical complications, the extent of resection, and patients’ survival data.

## Materials and methods

A cohort of patients who received surgical treatment for supratentorial gliomas between May 2011 and February 2016 at the Fondazione IRCCS Ca’ Granda Ospedale Maggiore Policlinico of Milano were retrospectively assessed for inclusion in this study.

### Inclusion criteria

The following criteria needed to be met for patient inclusion:
1) age superior to 18 years;
2) supratentorial gliomas;
3) tumor tissue available for histological and molecular diagnoses according to the WHO 2016 CNS brain tumors’ classification;
4) pre- and postoperative (obtained within 72 h) T2-weighted (w) images, T2-weighted fluid-attenuated inversion recovery (FLAIR) images, T1-weighted images before and after gadolinium intravenous administration, and diffusion-weighted images (DWI) available for radiological evaluation and volumetric analysis;
5) a tailored “small-craniotomy” as a surgical approach to the tumor;
6) surgical resection guided by intraoperative DTI and IONM;
7) completeness of the clinical chart during the review process by the Authors;
8) informed consent for participating in the study.

Patients suffering from recurrent tumors were also included in this study, in case the aforementioned inclusion criteria were met. These patients were classified as revision resections.

Ethical approval was waived by the local Ethics Committee of “Università degli Studi di Milano,” because of the retrospective nature of the study, and all the procedures being performed were part of routine care.

### Histopathological and molecular diagnoses

Tumor samples were formalin-fixed and analyzed by dedicated neuropathologists. Tumor grade and molecular profile were reported according to both the WHO 2016 and 2021 CNS brain tumor classifications (22).

MGMT was considered to be methylated when it showed more than 9% of promoter methylation as previously described (23).

### Clinical features

Baseline demographic features, preoperative KPS, length of the in-ward stay, postoperative neurological deficits, postoperative seizures’ rate—as their incidence during the in-ward stay and at follow-up (FU), destination at discharge, and neurological deficits still present at 3-month FU were collected from electronic clinical records and registers, and outpatient neuro-oncological evaluations.

The occurrence of a new postoperative neurological deficit was graded according to severity (severe or mild/moderate). The following deficits were considered severe: severe muscle strength deficit (grade 1–3 Medical Research Council Scale), hemianopia, and aphasia. All other neurological deficits were considered mild/moderate. Deficits still present at 3-month FU were considered definitive.

All patients underwent extensive pre- and postoperative neuropsychological testing using the battery of neuropsychological tests as reported previously (24–26).

### Radiological features

Preoperative MRI scans were performed on a 3T Philips Achieva (Best, the Netherlands) scanner, using a 32-channel phased-array head coil.

As part of daily care at our Institution, all patients suffering from intracranial tumors received preoperative and postoperative (within 72 h) brain MRIs. T2-weighted (w) images, T2w fluid-attenuated inversion recovery (FLAIR) images, T1w images before and after gadolinium intravenous administration, and diffusion-weighted images (DWI) were assessed for radiological feature extraction. Baseline radiological characteristics included tumor location—as affected cerebral lobe and side—contrast enhancement (CE), and preoperative and postoperative tumor volumes. Volumetric tumor measures were addressed by a trained resident neurosurgeon (G.F.) and reviewed by an experienced neurosurgeon (G.C.), both of them blinded to clinical information. We used the open-source software Horos (www.horosproject.org; Horos Project) for manual segmentation which allowed us to manually delineate, slice by slice, the tumor contour, thus creating a ROI dataset from which the tumor volume (cm^3^) was calculated. For CE tumors (i.e., ring-like contrast-enhancement pattern), regions of interest (ROIs) contoured CE boundaries. For tumors without CE or with patchy CE, ROIs contoured FLAIR alteration areas.

The extent of resection (EOR) was calculated as: [(preoperative tumor volume – postoperative tumor volume)/preoperative tumor volume] × 100 (27).

Postoperative DWI was checked for ischemic damage and postoperative pre-contrast T1w for hyperintense blood products.

### DTI imaging

Diffusion tensor imaging (DTI) acquisition included an axial single-shot spin-echo echo-planar imaging (EPI) sequence (TR 7,866 ms; TE 74 ms) with 64 diffusion-encoding directions. A diffusion gradient was applied using two b factors (0 and 1,000 s/mm^2^), with a sensitivity encoding reduction factor (SENSE) of 2 and a flip angle equal to 90°. Isotropic voxel dimensions of 2 × 2 × 2 mm^3^ were obtained by using a field of view of 225 × 225 mm^2^ and a matrix of 112 × 110. Seventy slices were obtained, with a thickness of 2 mm, with no gap. DTI data motion artifacts were adjusted using the installed software on the scanner. The total acquisition time was 8 min 47 s.

### Fiber tracking

Deterministic (28) fiber tracking based on DTI was performed using the iPlan 3.0 cranial planning software (Brainlab AG, Munich, Germany). DTI sequences were automatically corrected for head motion and eddy current distortions.

Fiber tracking of the corticospinal tract (CST) was obtained by placing a cubic box region of interest (ROI) along the precentral gyrus and a second one along the anterior pons. The SLF was reconstructed by placing a first ROI on the high-anisotropy region laterally to the central part of the lateral ventricle on a coronal section; the second ROI was placed on the peri-trigonal area at the level of the descending branch of the tract (1). To reconstruct the arcuate fasciculus (AF), three cubic seed ROIs were placed along the subcortical white matter of the opercular part of the inferior frontal gyrus, the inferior part of the precentral gyrus, the supramarginal gyrus, and the superior and medial temporal gyrus (19). To reconstruct the inferior frontal-occipital fasciculus (IFOF) and the uncinate fasciculus (UF), we constructed an ROI on the anterior floor of the external capsule at the junction of the frontal and temporal lobes, where the IFOF narrows in the section being contiguous to UF (29). To reconstruct the inferior longitudinal fasciculus (ILF), two ROIs were located along the subcortical white matter of the anterior temporal region and the occipital one (30). The frontal aslant tract (FAT) was reconstructed by placing an ROI on the opercular frontal cortex (inferior frontal gyrus) and a second one in the supplementary motor area (SMA) and pre-SMA cortices (superior frontal gyrus).

Fiber tracking was performed with a fractional anisotropy (FA) minimum threshold value of 0.15 (mainly 0.18–0.21), an angular threshold of 45° (not editable by the user), and a variable minimum fiber length of 30–50 mm.

### IONM methods

The armamentarium used for IONM has already been described elsewhere by our group (25, 31). The neurophysiological techniques included transcranial corkscrew electrodes for transcranial (TC) motor-evoked potentials (MEPs); DES with monopolar and bipolar probes for mapping motor and language functions; cortical strip for electrical stimulation of the cortex and cortical MEP monitoring; cortical strip for electrocorticography (ECoG); and electromyography (EMG) for continuous monitoring of motor functions.

The IONM was performed by dedicated neurophysiologists and technicians (FC, GA, LB, MV). The monitoring technique was performed as described previously (25, 31).

### Anesthesiological technique

In all patients, a total intravenous anesthesia protocol was performed with remifentanil and propofol, as previously reported. No curare or curare-like drugs were used in this series. In the case of awake craniotomy, the surgical opening was performed in an asleep–awake fashion with a laryngeal mask (5, 32).

### Surgical technique

#### IONM

The type of IONM was tailored to tumor location and patients’ characteristics, with regard to functional anatomy, as depicted by DTI-FT, and cognitive status, according to neuropsychological evaluation. EEG and EMG monitoring of motor response was used in all the cases, as described elsewhere (33).

In the revision process, we tried to simplify our IONM choices, as reported in **Table 1**. Briefly, tumors were classified according to the hemisphere and lobe being affected. Tumors in the dominant hemisphere were generally operated on awake to allow language and complex neurological function mapping and monitoring (asleep–awake technique). Tumors in the frontal or parietal lobe were simplistically considered tumors affecting the motor areas, and as previously described, IONM was decided based on the distance of the tumor from the motor cortex (M1) or CST [more than 3 cm or less than 3 cm (1, 15, 16, 34, 35)]. Gliomas located more than 3 cm away from the CST or M1 were monitored with continuous transcranial MEPs, with DES being applied mostly subcortically using a monopolar probe. Temporal and insular tumors were operated on with awake or asleep surgery according to their location in the dominant or non-dominant hemisphere, respectively. Non-dominant temporal tumors were operated on with transcranial MEP (TC MEP) monitoring and subcortical monopolar DES to check the distance from the midline structures while facing the medial part of the temporal lobe. Dominant temporal lobe tumors required bipolar DES at cortical and subcortical levels (similarly to dominant frontal lobe tumors) to map cortical language sites and important subcortical fiber bundles. Insular lobe tumors, considering their well-known surgical complexity, were generally approached using all the IONM armamentariums, especially when located in the dominant hemisphere (25). Occipital lobe tumors were treated with minimal IONM (TC MEP, rarely with subcortical monopolar DES), and their resection was generally guided by DTI with the reconstruction of the optic pathways (2).

**Table 1:** The scheme used for IONM choice according to the surgical procedure to be performed.

Lobe	Distance tumor/CST	Awake yes/no	MEP		DES - Cortical		DES - Subcortical		EcoG	EEG	EMG
			TC	Strip	Monopolar	Bipolar	Monopolar	Bipolar			
Frontal	> 3cm	no				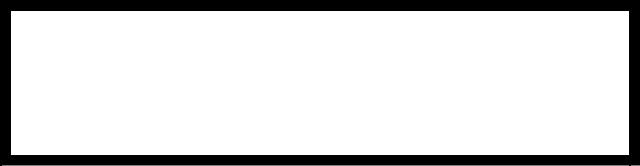		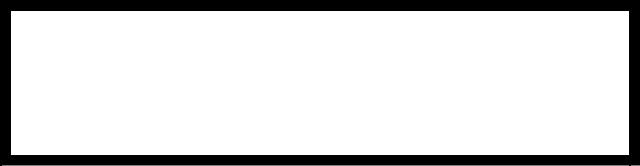			
	> 3cm	yes									
	< 3cm	no									
	< 3cm	yes									
Parietal	> 3cm	no				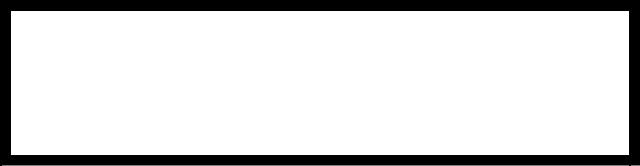		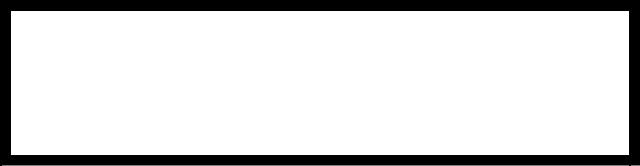			
	> 3cm	yes									
	< 3cm	no									
	< 3cm	yes									
											
Temporal		no		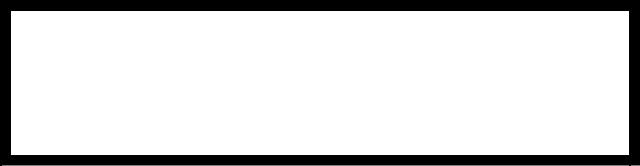	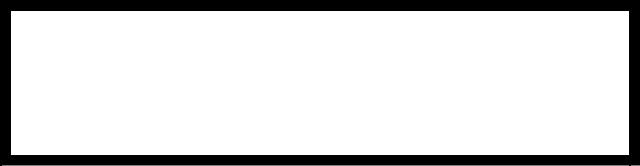	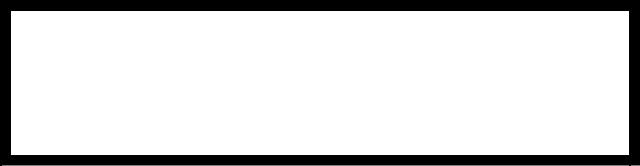		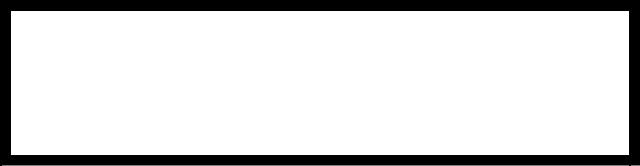			
		yes		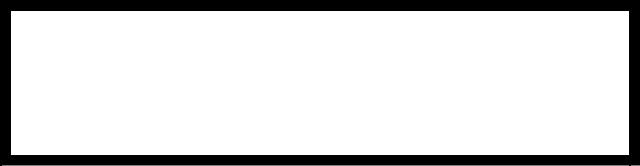							
Occipital				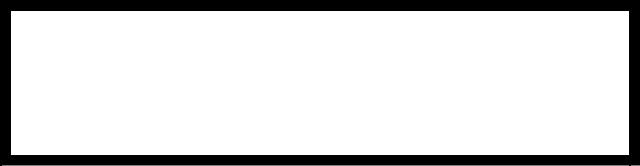	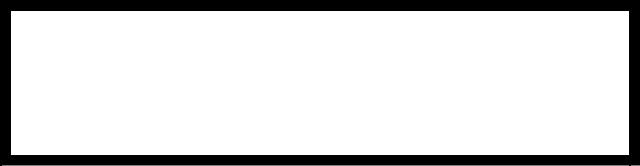	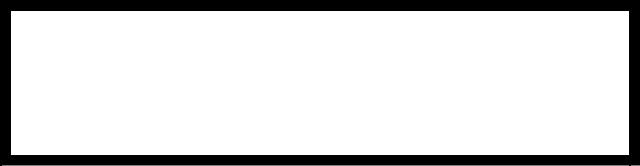		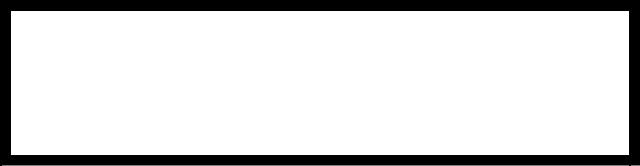			
											
Insular		no									
		yes									



 indicates that the neurophysiological technique was always used.


 indicates that the neurophysiological technique was NOT always used.

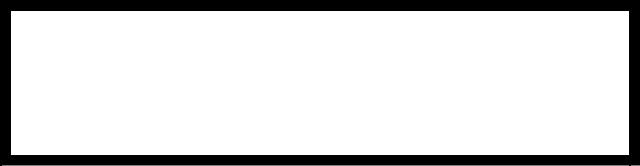
 indicates that the neurophysiological technique was never used.

#### Definition of small craniotomy

**Figure 1:**
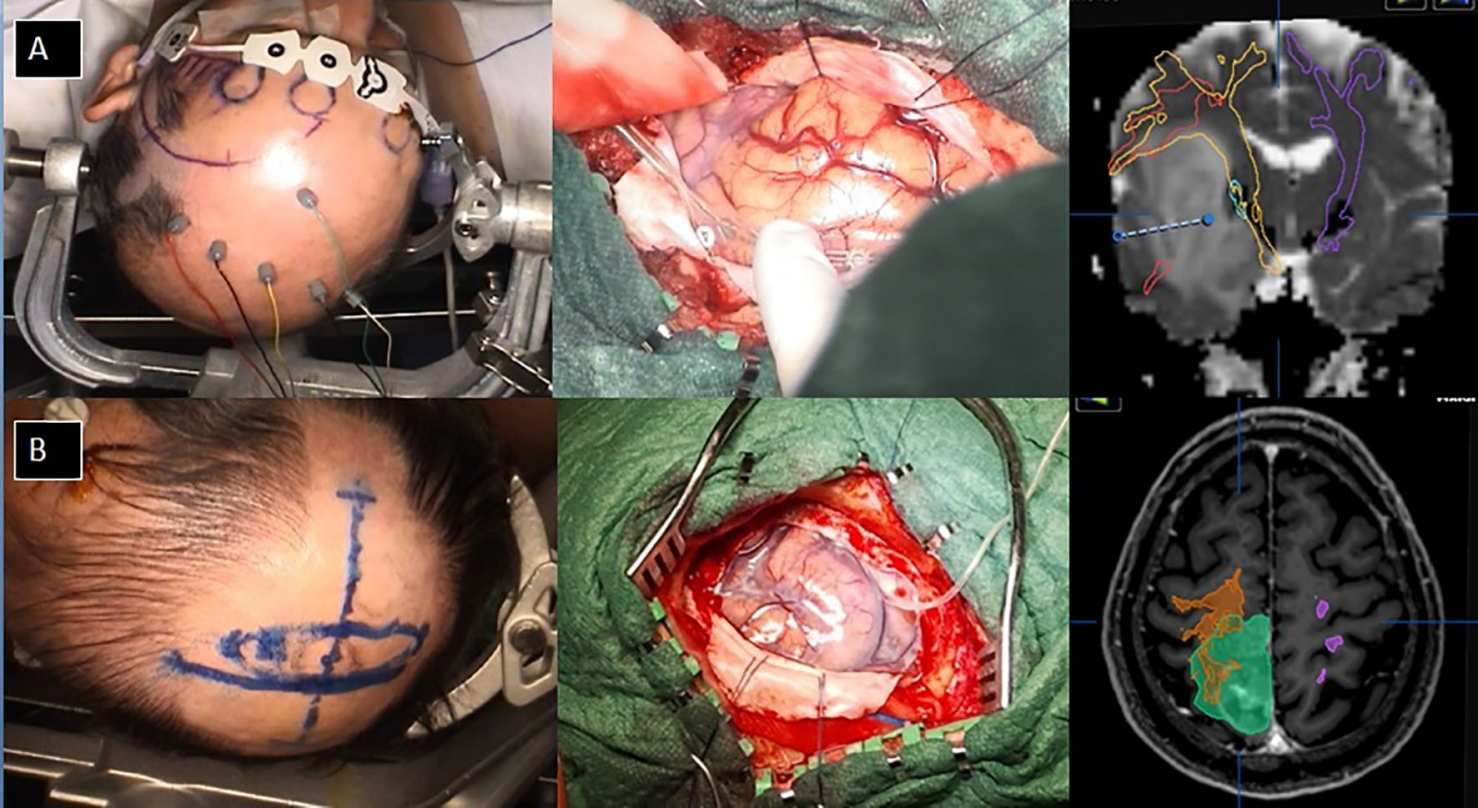
Examples of small craniotomy tailored on DTI and tumor projection to the cortex. **(A)** a case of an insula tumor with the intraoperative view and the neuronavigational planning; **(B)** a case of a parietal tumor with the intraoperative view and the surgical planning.

In our series, as “small craniotomy” we defined a craniotomy that did not extend beyond 1 cm from the maximal tumor diameters if the tumor was cortical or immediately subcortical or that did not extend beyond 1 cm of the maximal cortical tumor projections in case of a deep-seated location. In other words, no brain other than the strictly necessary to allow tumor resection was exposed. Some intraoperative pictures are available in **Figure 1**, showing a complete IONM despite the size of the craniotomy and surgical tailoring as aforementioned. The functional areas surrounding the tumor were not necessarily exposed.

#### Surgical resection of tumors close (≤3cm) to the motor area

In the case of Rolandic and peri-Rolandic gliomas (as defined by Berger in 2004 (16)), a small craniotomy was planned according to DTI and neuro-navigation (Brainlab, Munich, Germany). The craniotomy was performed over the tumor aiming to expose the lesion and the strictly necessary cortex, which, in this case, could or could not include M1 (**Figure 1**). After opening the dura, we mostly employed a monopolar low-intensity and high-frequency (HF) electrical stimulation to identify the cortical (M1) and subcortical motor pathways. When a stable cortical motor response was achieved, the intensity of stimulation was further decreased to the threshold (36), to enhance M1 detection. If increased focality/specificity of cortical stimulation was needed to discriminate M1 from premotor or sensitive areas, a bipolar low-frequency (LF) electrical stimulation was also exploited.

A cortical strip with four to six platinum contacts (Integra, Princeton, NJ, USA) was placed on the motor cortex to allow monitoring of the motor function throughout the procedure. In case the motor cortex was not fully exposed, then the cortical strip was made to slide under the dura with care. After the localization of M1, then brain mapping was performed around the tumor to exclude the presence of peritumoral motor functioning areas. After the required corticectomy was performed, continuous monitoring of the motor pathway was achieved with cortical MEPs with a cortical or subdural strip. At this point, DTI was employed to help in the neurophysiological definition of the CST. Subcortical resection close to the motor pathways was performed alternating mapping with the monopolar probe and the aspiration (eventually with CUSA) of tumoral/peritumoral areas as far as it was considered safe, according to brain/tumor visual appearance. Tumor resection was continued until a positive response at the intensity of 2–3 mA with the monopolar probe was obtained.

#### Surgical resection of tumors in frontal or parietal lobe not close to the cortical motor areas

In the case of gliomas located in the frontal or parietal lobe but not close to the Rolandic area, the craniotomy was placed right over the tumor (or over its cortical projections), with minimal brain exposure. In this way, the motor area was not generally exposed and positioning cortical electrodes under the dura and over the motor area strip (for cortical MEP monitoring) was in most cases not possible. For this reason, before surgery, corkscrew electrodes were placed on the scalp, in the skin area that was over the projection of the motor area. Occasionally, DTI reconstruction of the CST was used to help the technician in the definition of the area and correct positioning of the corkscrew electrodes. Continuous TC MEPs were monitored during surgery. After exposure of the lesion, the resection started with a corticectomy (when necessary) and subsequent dissection of the tumor from the normal brain parenchyma using Penfield dissectors and CUSA from time to time. During the resection of the deeper part of the tumor, monopolar stimulation was used to assess the distance from the CST. During surgical resection of larger lesions, phenomena of brain shift and pneumocephalus may decrease TC MEP responses. At this point, positioning a cortical strip for cortical MEP monitoring became generally possible.

#### Surgical resection of tumors in the temporal or occipital lobe in the non-dominant hemisphere

In the case of gliomas located in non-dominant temporal and occipital lobes, the surgical resection was carried out similarly to what was done for frontal or parietal lobe tumors located >3 cm away from the Rolandic area. Essentially, motor monitoring was performed using TC MEP with corkscrew electrodes and monopolar DES was done subcortically. In addition to DTI for the CST, in occipital lobe tumors, the resection was planned and carried out taking into consideration the DTI reconstruction of the optic pathways, as described elsewhere (2).

#### Surgical resection of tumors in the dominant hemisphere operated with asleep–awake technique

In the case of tumors, located in the dominant hemisphere affecting frontal and temporal lobes and, at times, parietal and occipital lobes, the asleep–awake technique was used for surgical resection. As extensively published, the awake technique is the most reliable and safest technique to preserve complex functions in patients with brain tumors in the dominant hemisphere. In particular, it allows intraoperative testing of the patients with specific tasks for language expression and comprehension as well as customized tasks for the preservation of other skills such as music, cognitive functions, and social recognition. The authors applied awake surgery in all the cases of LGG affecting the dominant hemisphere and in most cases of HGG. The utility of the awake technique in HGG has been extensively debated (37, 38), and, in this series, it was used in selected HGG patients after multidisciplinary evaluation by neurosurgeons, neuropsychologists, and anesthesiologists.

**Figure 2:**
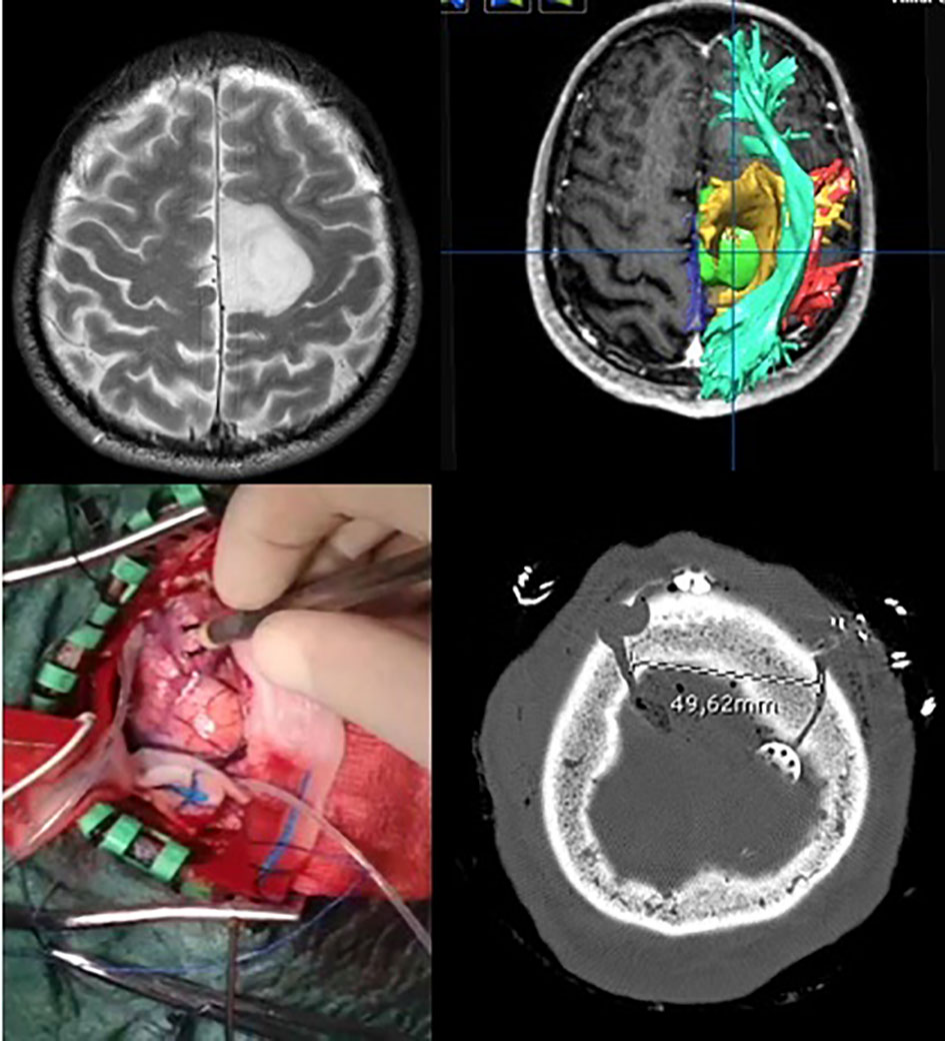
Example of awake surgery for a left frontal low-grade glioma performed with a targeted small craniotomy sized about 4.5 cm.

In the asleep–awake setting, a tumor-targeted craniotomy was performed as well (**Figure 2**). Then, the patient was awakened, the laryngeal mask was removed, and once the patient was collaborative, the brain mapping for language and other functions was performed using bipolar DES (Penfield’s technique). When the lesion was in proximity to the motor area, then the MEP monitoring was performed using a cortical strip; on the contrary, when the lesion was not close to the Rolandic area, TC MEP was employed. Bipolar DES was applied also subcortically, using a generally slight increase in the 60-Hz currents (10%–20%). Monopolar DES was used for motor mapping at both the cortical and subcortical levels.

DTI was used to plan the craniotomy and to help in the definition of the lesion as it regards its relationship with the CST but also the main language fiber bundles. In particular, as described elsewhere, the IFO and the arcuate fasciculus were reconstructed using DTI and defined intraoperatively by DES.

#### Surgical resection of tumors located in the insular lobe

**Figure 3:**
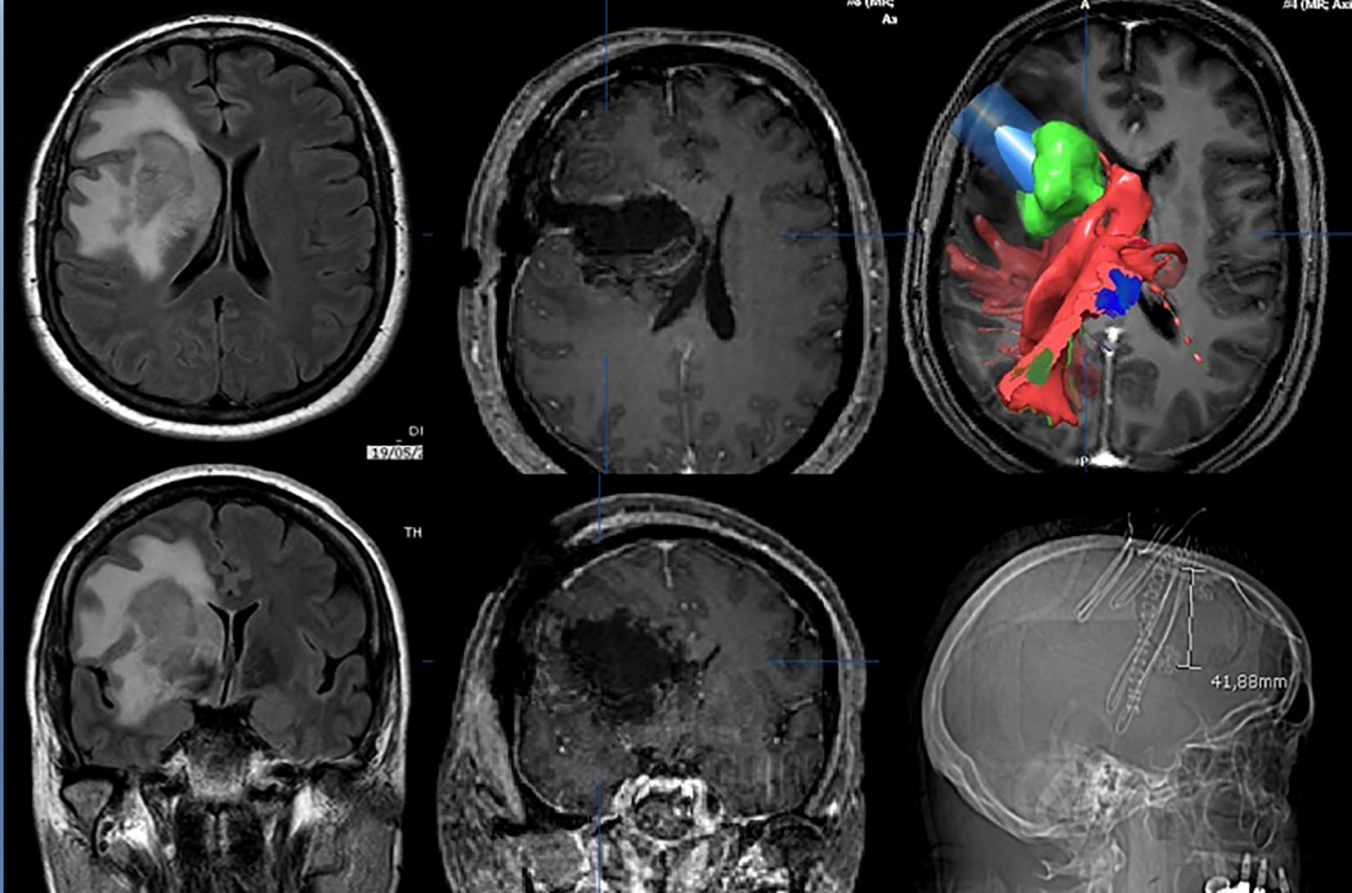
Fronto-opercular approach to an insular high-grade glioma targeted according DTI and IONM.

The insula is located deeply in the Sylvian fissure, and the resection of these gliomas is among the most challenging. Given these premises, it is clear that the greatest efforts were made preoperatively to get complete DTI reconstruction of the important white matter tracts and intraoperatively to obtain a reliable and complete IONM (**Figure 3**).

In addition to EEG, TC MEPs were always applied. The most commonly used approach to the insular lobe was the trans-opercular approach. On the non-dominant side, this approach required cortical brain mapping (using monopolar DES) to avoid the motor area of the face, while in the dominant hemisphere awake surgery was necessary to avoid injury to the language function. The technique in the dominant hemisphere was similar to the one adopted for dominant frontal or parietal gliomas in terms of brain mapping and monitoring of language functions. Nevertheless, the technical difficulties due to perforating branches of the middle cerebral artery, the length of surgery, and the deeper location of these tumors require higher technical skills than other gliomas.

### Intraoperative variables

Data concerning surgical procedures were retrieved from digital operative registers. The following variables of interest were collected: type of anesthesia (asleep vs. asleep–awake or asleep–awake–asleep), type of intraoperative neurophysiological monitoring (IONM), surgical procedure duration, and occurrence of intraoperative seizures.

The use of the following IONM techniques was analyzed and collected: transcranial electrical stimulation (TC) with motor-evoked potentials (MEPs) and somatosensory-evoked potentials (SSEPs), electroencephalography (EEG), electrocorticography (ECoG), direct electrical stimulation (DES), monopolar stimulation for subcortical mapping, and bipolar stimulation for cortical mapping.

Intraoperative seizures were classified according to their clinical manifestation—as electrical or symptomatic—and the treatment needed to stop them—cold water irrigation or drugs (32).

In the case of recurrent tumor resection, it was recorded if an extension of the previous mini-craniotomy was required.

### Outcome measures

Three outcome measures were assessed for the patients: overall survival (OS), progression-free survival (PFS), and rate of definitive neurological deficits (deficits that did not regress at the 3-month follow-up). The postoperative tumor volume and EOR were identified as the main surgical outcomes.

OS was defined as the time from surgery to death. PFS was defined as the time from surgery to tumor progression. We referred to RANO criteria for gliomas to assess disease progression (39).

Patients who did not experience death or disease progression were censored at their last scan date, during OS and PFS analyses.

### Statistical analyses

Variables of interest were reported and compared as follows:

**•** Frequencies were reported as a percentage and compared by chi-square/Fisher exact test;
• Continuous variables were checked for normal distribution through skewness, kurtosis, and Shapiro–Wilks normality test;
• The continuous normally distributed variables were reported as mean ± standard deviation and compared through Student’s t-test or variance analyses.
• The continuous skewed distributed variables were reported as median (interquartile range, IQR) and compared *via* Mann–Whitney U-test and Kruskal–Wallis test.

A multiple linear regression model (using the Enter method) was settled to assess the predicting value of explanatory variables on the surgical predicted outcome (postoperative volume and EOR). The explanatory variables were chosen based on recent literature as well as on the statistical significance of the univariate analysis. Multicollinearity was assessed at each step through tolerance and the variance inflation factor (VIF). Residuals were checked for normality.

OS and PFS as a function of time were calculated by the Kaplan–Meier method.

Univariate analysis of risk factors influencing PFS and OS was assessed by the proportional hazard regression model. Variables associated with PFS and OS in univariate analyses (p < 0.05) were included in the multivariate Cox proportional hazard regression model. All statistical analyses were performed using IBM SPSS version 25.0, International Business Machines Corp, New York, USA.

## Results

In the period between May 2011 and February 2016, 265 patients received surgical treatment for supratentorial gliomas. One hundred and three patients met the inclusion criteria for the study. Of these, 21 patients received a revision resection. The overall median follow-up was 19 months (range from 3 to 108 months). Follow-up ended in November 2021. The main variables of interest are summarized in **Table 2**.

**Table 2:** Clinical and histological features of our series.

Patient Population	103
First Resection	82
Revision Surgery	21
	
WHO 2016	
Grade IV	65
Grade III	19
Grade II	19
	
WHO 2021	
oligodendroglioma	14
astrocytoma	9
GBM -like	80
	
Age	54 ± 14
	
Sex (male)	61
	
Preoperative KPS (median)	80
	
Length of in-ward Stay (days)	7 (IQR 4)
	
Intraoperative Symptomatic Seizures	7
	
Post-operative Seizures	9
	
New Postoperative Deficits	31
	
3-month Follow-up Deficits	3

### Histopathological tumor features

According to WHO 2016 brain tumor classification (22), 65 patients suffered from WHO grade IV gliomas (63%), 19 patients from WHO grade III gliomas (19%), and 19 patients from WHO grade II gliomas (19%). WHO grade III gliomas included 15 astrocytomas (6 IDH 1 or 2 mutated) and four oligodendrogliomas (all IDH 1/2 mutated). WHO grade II tumors embedded eight astrocytomas (2 IDH 1 or 2 mutated) and 11 oligodendrogliomas (all IDH 1 or 2 mutated).

The MGMT gene promoter methylation showed superiority at 9% in 55 cases (53%).

All the patients with WHO grade IV tumors received chemoradiotherapy according to the Stupp protocol except eight patients (6 received only temozolomide, 1 fotemustine, and 1 did not receive any adjuvant treatment). All the patients with WHO grade III tumors received chemoradiotherapy except three patients (2 received only temozolomide and 1 did not receive any adjuvant treatment). Among patients with WHO grade II tumors, only four patients received adjuvant chemoradiotherapy.

According to WHO 2021 brain tumor classification, we identified 14 patients with a molecular oligodendroglioma profile (IDH1 or 2 mutations, and 1p–19q chromosomal arm heterozygosity loss), of which 10 are WHO grade II and four WHO grade III. Nine patients showed a molecular astrocytoma profile (IDH1 or 2 mutations, with intact 1p–19q), among which three showed WHO grade II histological features and six with WHO grade III histological characteristics. Eighty patients showed glioblastoma (GBM)-like features: IDH1 and 2 wild types (wt) and imbalance of chromosome 7 and chromosome 10q loss of heterozygosity, or telomerase reverse transcriptase (TERT) promoter mutation, or epidermal growth factor receptor (EGFR) amplification (40). All the patients with GBM molecular features received chemoradiotherapy except 12 patients (7 received only temozolomide and 5 did not receive any adjuvant treatment). Among patients with astrocytoma molecular features, five patients received adjuvant chemoradiotherapy. Among patients with oligodendroglioma molecular features, four patients received adjuvant temozolomide.

### Patients population

The mean age was 54 ± 14 years. Sixty-one patients (59%) were men. The mean preoperative KPS was 80. One-way ANOVA showed a statistically significant variance among age means of the different WHO tumor grades (F = 4; p = 0.002) as well as among the three molecular subtypes (F = 7; p = 0.001). *Post-hoc* analyses using the LSD method revealed greater age means among WHO grade IV glioma carriers (58 ± 12 years), compared to others (45 ± 15 years), and among patients with GBM and GBM-like molecular profiles (57 ± 13 years), in comparison to those with oligodendroglioma (46 ± 14 years) and astrocytoma (43 ± 16 years) molecular features.

Chi-square analyses revealed a higher incidence of preoperative KPS rates inferior to 80 among glioblastoma carriers (51%), in comparison to those suffering from lower-grade gliomas (7%–33%), and among patients with a GBM-like molecular profile (46%) when compared with both of those with astrocytoma (0%) and oligodendroglioma (7%).

### Clinical characteristics

The median length of the in-ward stay was 7 (IQR 4) days. Nine patients (9%) experienced seizures during the in-ward stay, requiring an antiepileptic drug increase and/or multidrug association. All but two of these patients experienced partial epileptic events. One patient suffered from a generalized seizure, while another from a delayed postoperative awakening.

Postoperative neurological deficits occurred in 31 patients, of which 14 were severe. Ten patients showed an improved neurological status at postoperative evaluations. At the 3-month FU, only three neurological deficits were still present (3%). Seventeen patients (17%) were discharged to a rehabilitation facility.

### Intraoperative variables

Thirty-three patients underwent craniotomy under asleep–awake anesthesia. All patients underwent mini-craniotomy with at least EEG, EMG, and TC MEP as IONM. In 77 patients (75%), ECoG was used. DES was exploited in 51 patients (50%). Cortical mapping required monopolar and bipolar stimulation in 24 cases (23%). Subcortical monopolar stimulation was exploited in 33 cases (32%).

Fourteen patients (13.6%) experienced intraoperative seizures, of which eight (7.7%) were only electrically evident. Seven patients (6,8%) experienced intraoperative symptomatic (partial) seizures which required cold irrigation as a unique treatment, while two patients (2%) experienced symptomatic (partial) seizures that required pharmacological treatment.

The exact Fisher chi-square test revealed a statistical correlation between the incidence of intraoperative seizures and the employment of bipolar stimulation for brain cortical mapping (8/24 patients, p = 0.003).

Among the 21 revision resection cases, only one needed an extension of the previous craniotomy.

### Volumetric analyses

The median preoperative volume was 24 cc (IQR 42). The median postoperative volume was 0 cc (IQR 3). The median EOR among all gliomas was 100% (IQR 10%).

The following explanatory variables were put in the multiple linear regression model to predict postoperative volume values: preoperative volume, WHO 2016 gliomas’ classification type, and tumor side and location (as affected encephalic lobe). Increasing preoperative volume (R^2^ = 0.5; b = 0.15; ES = 0.03; ß = 0.5; p = 0.0001), WHO grade II IDH wt gliomas (R^2^ = 0.5; b = 13.7; ES = 3; ß = 0,3; p = 0.0001), and insular gliomas (R^2^ = 0.5; b = 5; ES = 2; ß = 0.2; p = 0.023) were related to greater postoperative tumor volumes.

The same regression analysis was conducted to predict EOR: WHO grade II IDH wt (R^2^ = 0.5; b = -24; ES = 5; ß = -0.4; p = 0.0001) and mut (R^2^ = 0.5; b = -14; ES = 3; ß = -0.3; p = 0.0001) gliomas and WHO grade III IDH mut (R^2^ = 0.5; b = -15; ES = 3.6; ß = -0.3; p = 0.0001), insular (R^2^ = 0.5; b = -11; ES = 3; ß = -0.3; p = 0.0001), and thalamic (R^2^ = 0.5; b = -10.5; ES = 6; ß = -0.1; p = 0.0001) gliomas were related to lower EOR.

**Figure 4:**
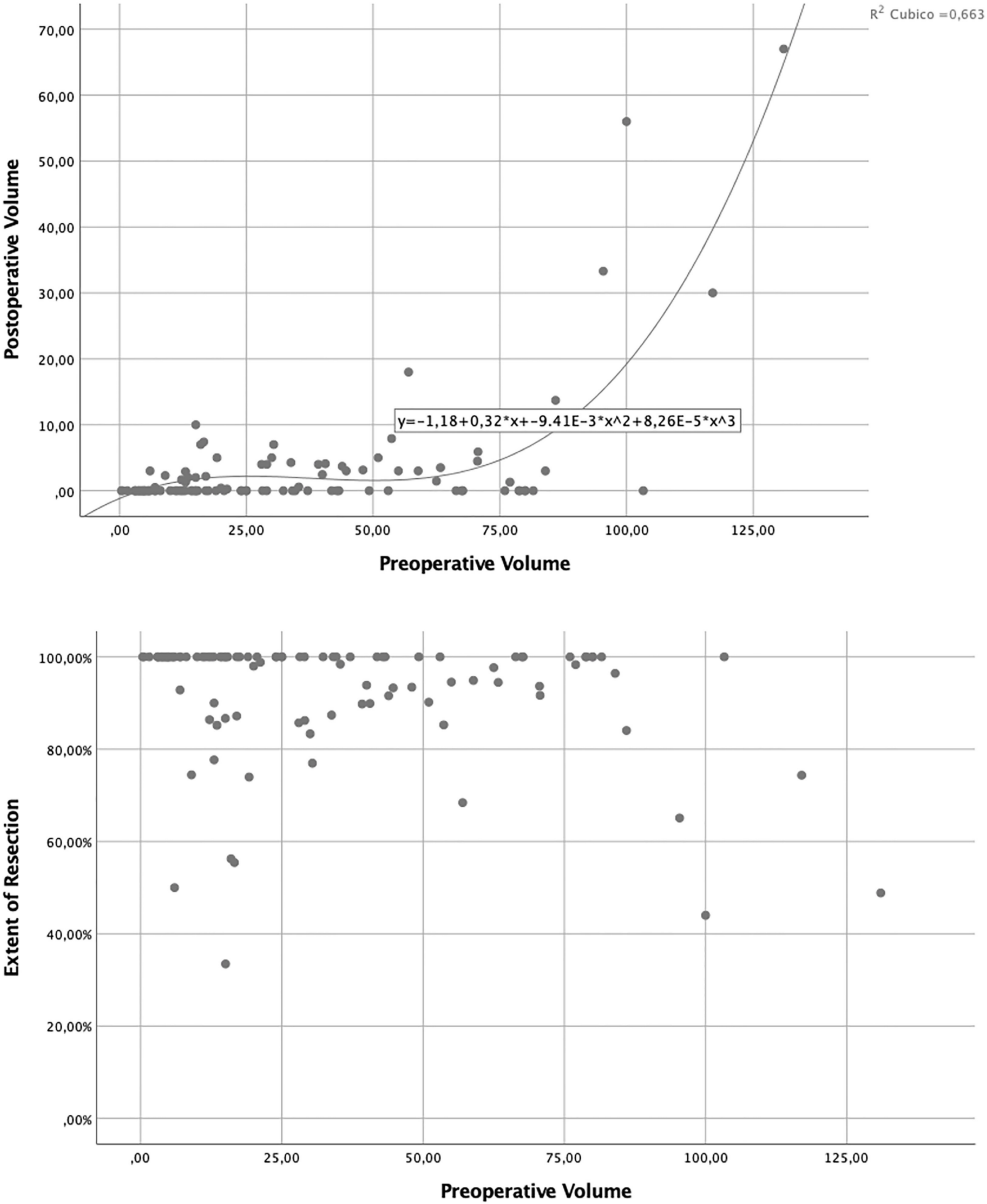
Volumetric analysis showing relations between preoperative tumor volume, residual tumor volume, and extent of resection.

Postoperative volumes resulted to be predicted by the preoperative ones, as a function of a cubic regression line (R2: 0.7; **Figure 4**).

### First resection

Preoperative volume medians of the different WHO tumor grades and molecular subtypes were compared, without any difference between groups being shown. The WHO grade II IDH1/2wt tumors had the largest preoperative volume median (38 cc, IQR 70), while the WHO grade III IDH1/2wt gliomas had the minimum preoperative volume median (15 cc, IQR 21).

**Figure 5:**
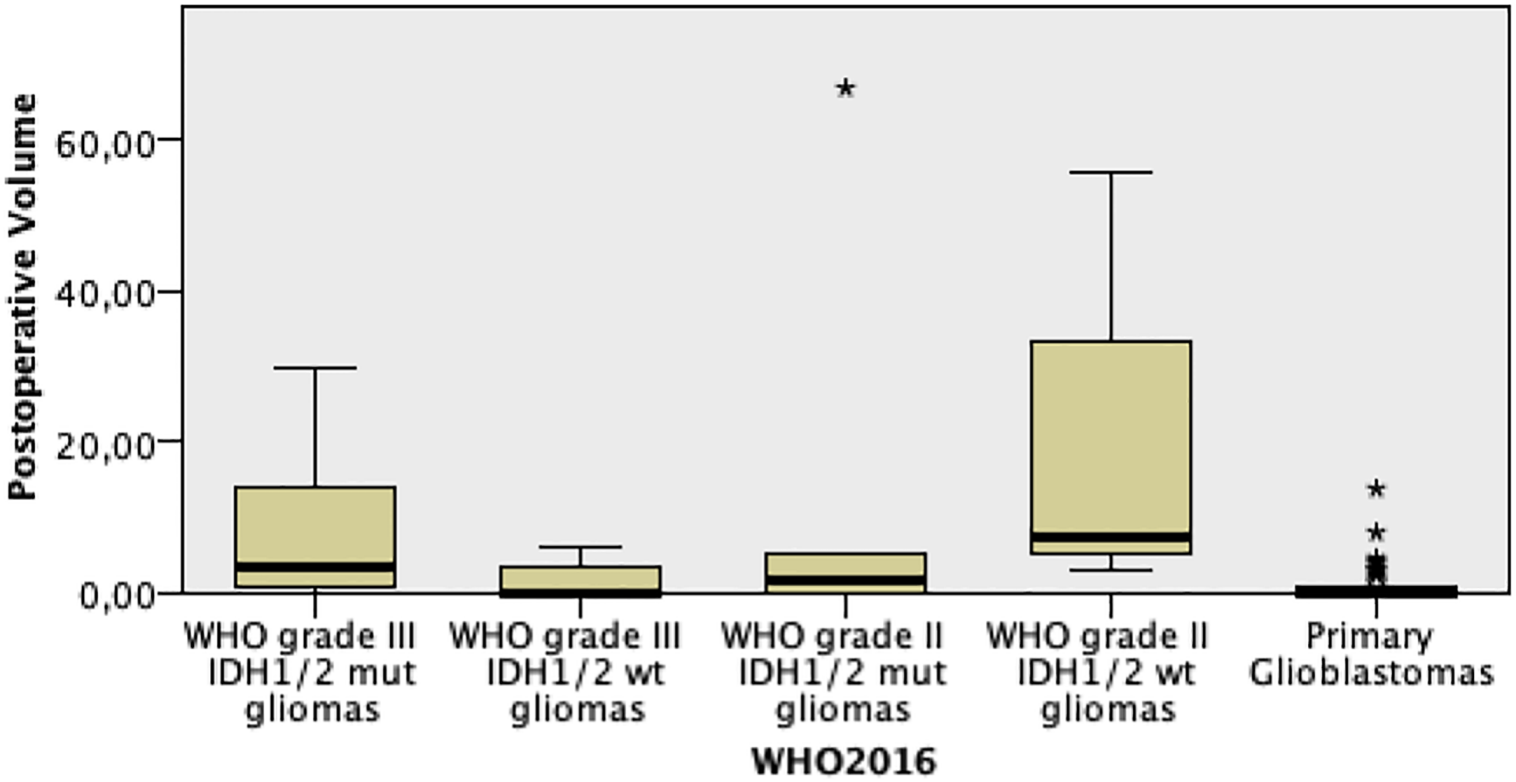
Postoperative volume comparison among the different WHO 2016 grade gliomas. * means outlayer.

Glioblastomas and WHO grade III IDH wt gliomas showed the lowest postoperative volume medians, equal to 0 cc, reaching statistical significance at medians’ comparison (p = 0.0001, **Figure 5**).

**Figure 6:**
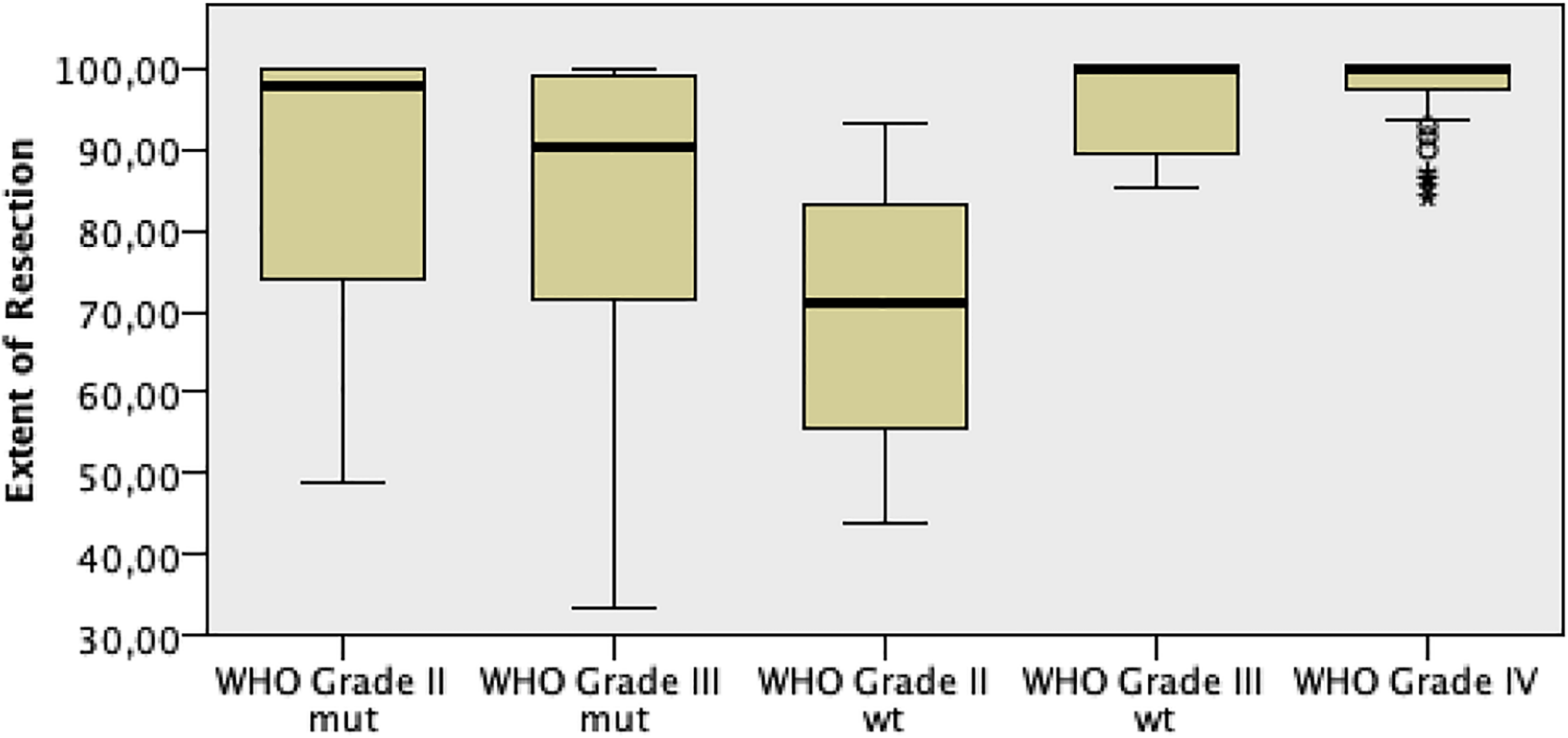
EOR comparison among the different WHO 2016 grade gliomas.

**Figure 7:**
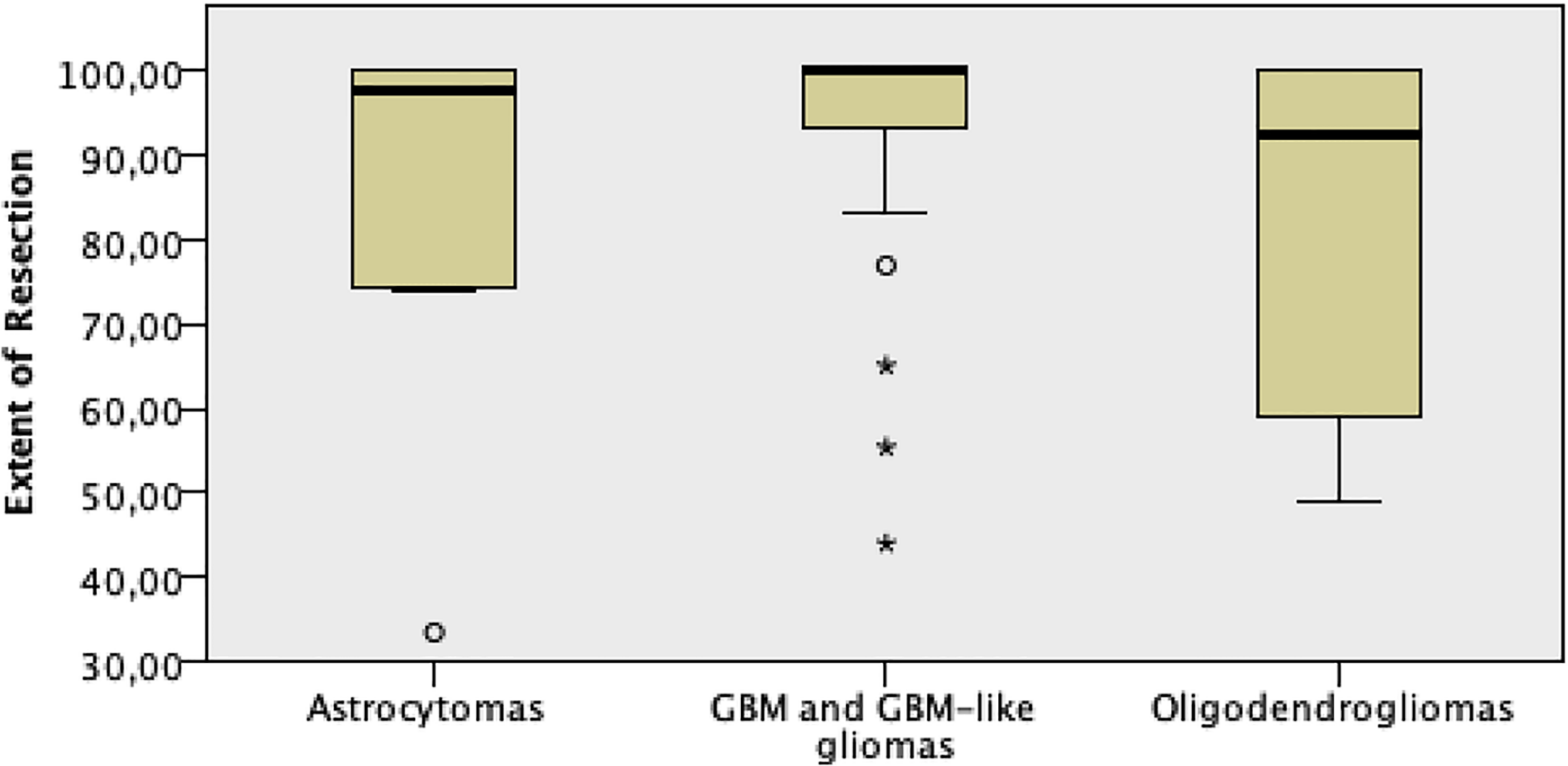
EOR comparison among the different molecular subtype gliomas.

EOR medians resulted to be statistically different among WHO tumor grades, with the highest EOR values among grade IV gliomas (p = 0.0001, **Figure 6**). When EOR medians were compared among the three molecular subtypes, no statistically significant differences were noted, with a positive trend of higher EOR rates among GBM and GBM-like gliomas (p = 0.067, **Figure 7**).

### Revision surgery

The preoperative and postoperative volume medians’ comparison among the different WHO tumor grades and molecular subtypes in revision surgery cases did not reach statistical significance in Kruskal–Wallis tests (respectively p = 0.138 and p = 0.112).

The comparison of EOR medians among different WHO grades as well as among the three different molecular subtypes demonstrated significantly higher rates among grade IV gliomas (p = 0.038) and GBM/GBM-like gliomas (p = 0.036).

### Overall survival analyses

The log-rank test using the Kaplan–Meier method indicated a statistically significant OS difference among all WHO grades (p = 0.0001). The median OS for WHO grade III IDH1/2mt gliomas was 74 months, for WHO grade III IDH1/2wt gliomas 20 months, and for WHO grade IV gliomas 14 months. For WHO grade II IDH1/2mt and wt gliomas, the median OS was not reached.

A statistically significant difference was also demonstrated among the OS of the three molecular groups (p = 0.0001). The median OS for GBM and GBM–like gliomas was 15 months. The median OS for astrocytomas and oligodendrogliomas was not reached.

The log-rank test using the Kaplan–Meier method was also employed to assess PFS, showing a statistically significant difference among all WHO grades (p = 0.0001). The median PFS for the WHO grade III IDH1/2wt gliomas was 12 months and for the WHO grade IV gliomas 10 months. For the WHO grade II IDH1/2mt and wt gliomas as well as for the WHO grade III IDH1/2mt ones, the median PFS was not reached. As for the OS, a statistically significant difference was noted among the three molecular groups (p = 0.0001). The median PFS for GBM and GBM–like gliomas was 12 months. For astrocytomas and oligodendrogliomas, the median PFS was not reached.

### First resection

Survival analyses were repeated on the only first resected gliomas.

**Figure 8:**
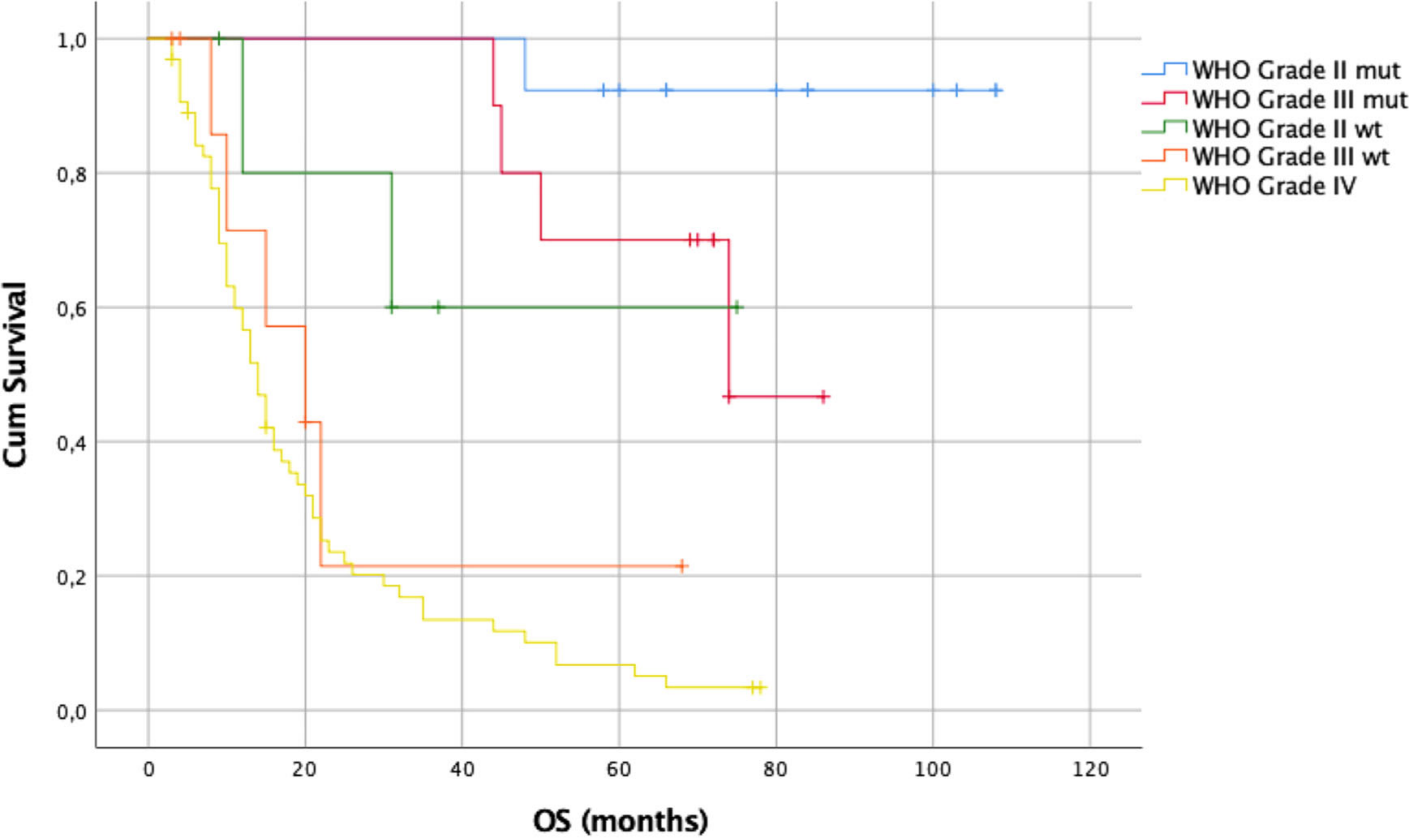
Kaplan–Meier curves of OS of patients at first resection.

The log-rank test using the Kaplan–Meier method showed a statistically significant difference among the OS of all WHO grades (p = 0.0001, SM 11A). The median OS for WHO grade III IDH1/2mt gliomas was 74 months, for WHO grade III IDH1/2wtgliomas 15 months, and for WHO grade IV gliomas 14 months. For WHO grade II IDH1/2mtand wt gliomas, the median OS was not reached (**Figure 8**).

The 1-, 2-, and 5-year OS rates were 100%, 100%, and 90% for WHO grade II IDH1/2mt gliomas, 67%, 67%, and 17% for WHO grade II IDH1/2wt gliomas, 100%, 100%, and 75% for WHO grade III IDH1/2mt gliomas, 37.5%, 12.5%, and 12.5% for WHO grade III IDH1/2wt gliomas, and 58.8%, 25%, and 4% for WHO grade IV gliomas.

**Figure 9:**
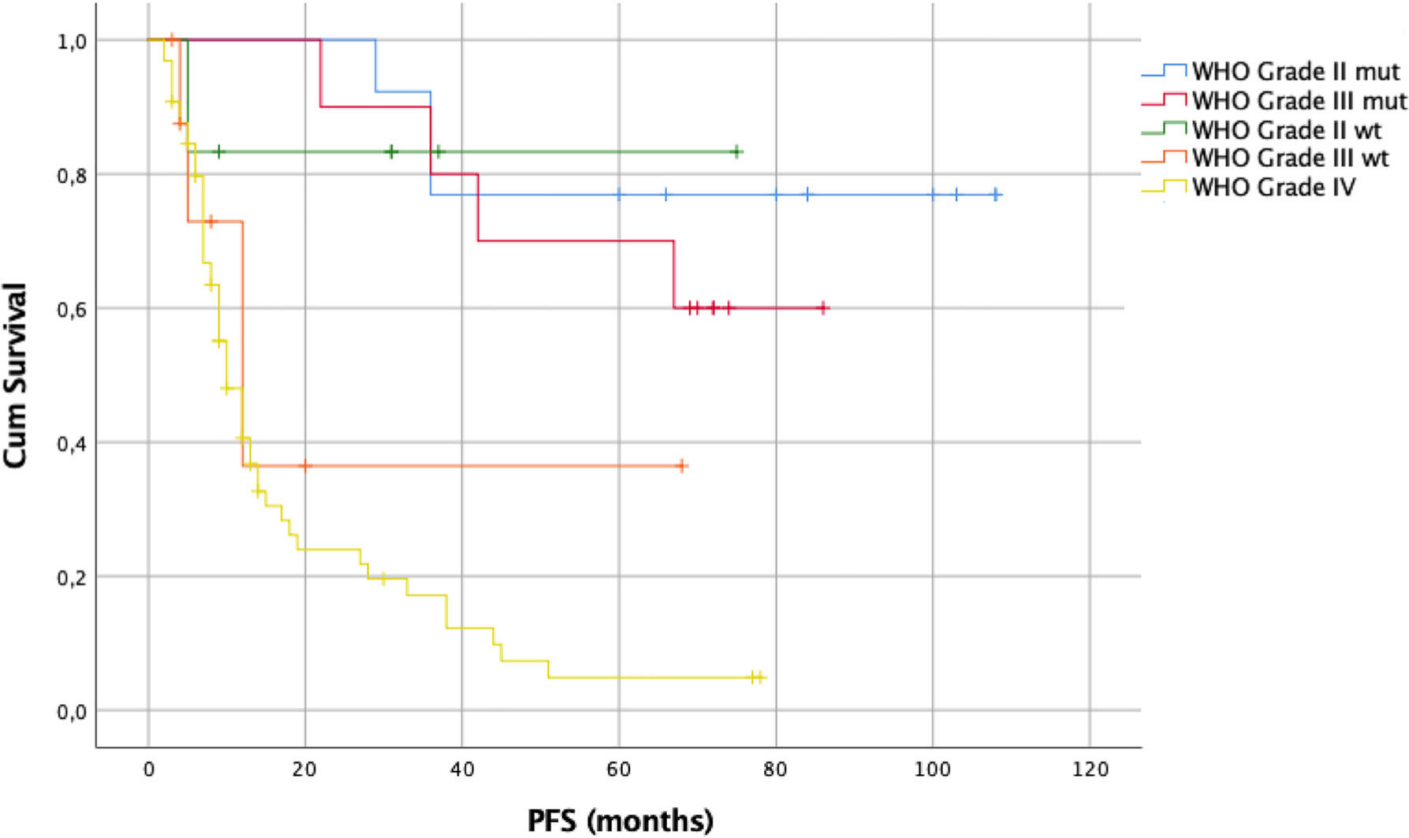
Kaplan–Meier curves of PFS of patients at first resection.

As for OS, the log-rank test using the Kaplan–Meier method was used to assess PFS, showing a statistically significant difference among all WHO grades (p = 0.0001, SM 11B). The median PFS for the WHO grade III IDH1/2wt gliomas was 12 months and for the WHO grade IV gliomas 9 months. For the WHO grade II IDH1/2mtand wt gliomas as well as for the WHO grade III IDH1/2mt ones, the median PFS was not reached **Figure 9**.

The 1-, 2-, and 5-year PFS rates were respectively 100%, 100%, and 90% for WHO grade II IDH1/2mt gliomas, 67%, 67%, and 17% for WHO grade II IDH1/2wt gliomas, 100%, 100%, and 75% for WHO grade III IDH1/2mt gliomas, 37.5%, 12.5%, and 12.5% for WHO grade III IDH1/2wt gliomas, and 39%, 20%, and 4% for WHO grade IV gliomas.

**Figure 10:**
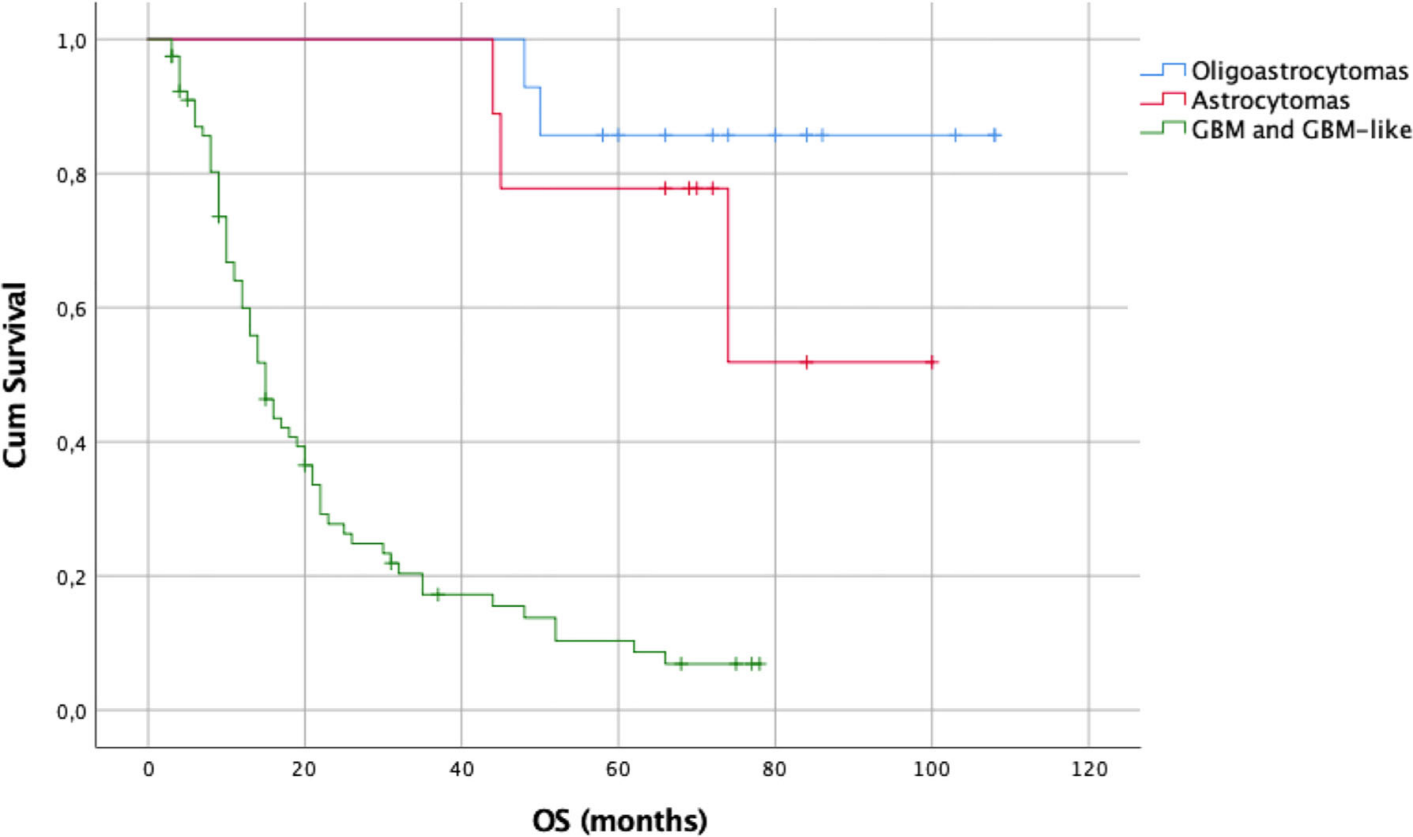
Kaplan–Meier curves of OS according to glioma molecular subtypes at first resection.

Concerning molecular subtypes, the log-rank test using the Kaplan–Meier method showed statistically different OS among the three molecular groups (p = 0.0001 **Figure 10**). The median OS for GBM and GBM–like gliomas was 15 months. The median OS for astrocytomas and oligodendrogliomas was not reached.

The 1-, 2-, and 5-year OS rates were respectively of 60%, 28%, and 10% for GBM and GBM–like gliomas, 100%, 100%, and 78% for astrocytomas, and 100%, 100%, and 88% for oligodendrogliomas.

**Figure 11:**
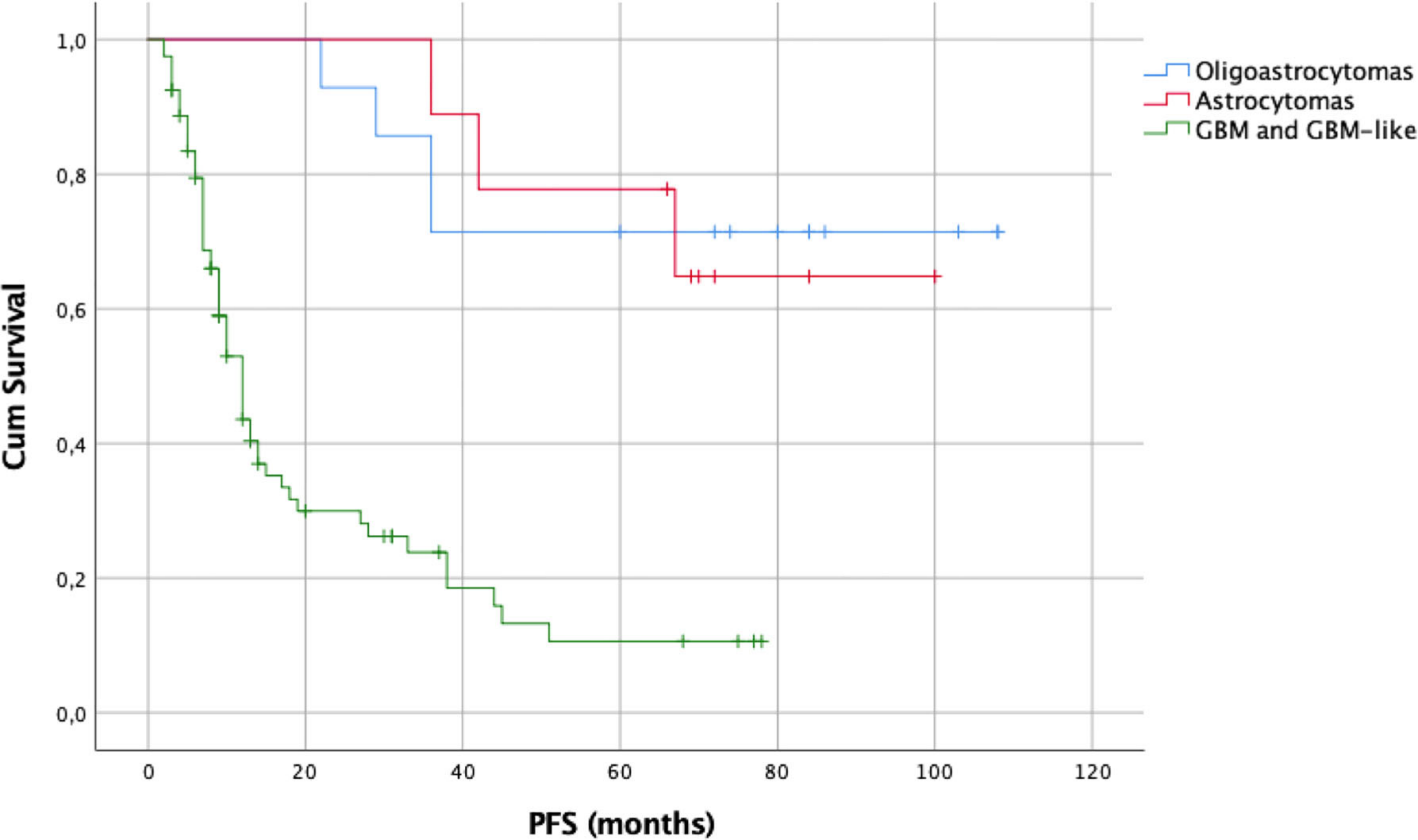
Kaplan–Meier curves of PFS according to glioma molecular subtypes at first resection.

The survival analysis was repeated to address PFS, showing a statistically significant difference among the three molecular groups (p = 0.0001 **Figure 11**). The median PFS for GBM and GBM–like gliomas was 10 months. For astrocytomas and oligodendrogliomas, the median PFS was not reached.

The 1-, 2-, and 5-year PFS rates were 42%, 23%, and 6% for GBM and GBM–like gliomas, 100%, 100%, and 78% for astrocytomas, and 100%, 100%, and 88% for oligodendrogliomas.

Age, affected side, preoperative KPS, and WHO 2016 gliomas’ classification affected PFS and OS at univariate analysis using the proportional hazard regression model. In the Cox proportional hazard multivariate regression model, only preoperative KPS (p = 0.006) and WHO 2016 grade II (p = 0.009) and III (p = 0.021) IDH1/2 mut gliomas confirmed a statistical correlation with the overall PFS and OS.

### Revision surgery

The survival analyses through the log-rank test using the Kaplan–Meier method were not performed on the revision surgery group due to the limited sample size.

The 1-, 2-, and 5-year OS rates were 100%, 100%, and 75% for WHO grade II IDH1/2mt gliomas; 100%, 100%, and 50% for WHO grade III IDH1/2mt gliomas; 100%, 0%, and 0% for WHO grade III IDH1/2wt gliomas; and 50%, 7%, and 0% for WHO grade IV gliomas.

The 1-, 2-, and 5-year PFS rates were 100%, 100%, and 50% for WHO grade II IDH1/2mt gliomas, 100%, 50%, and 50% for WHO grade III IDH1/2mt gliomas, 100%, 0%, and 0% for WHO grade III IDH1/2wt gliomas, and 42%, 7%, and 0% for WHO grade IV gliomas.

The 1-, 2-, and 5-year OS rates were of 53%, 7%, and 0% for GBM and GBM–like gliomas and 100%, 100%, and 67% for oligodendrogliomas.

The 1-, 2-, and 5-year PFS rates were of 47%, 7%, and 0% for GBM and GBM–like gliomas and 100%, 84%, and 50% for oligodendrogliomas.

## Discussion

In the last 20 years, the importance of white matter tracts has been rediscovered in neurosurgery (3, 6, 15, 35, 41, 42). Before, it was a common belief that the preservation of cortical functional areas was the most important surgical target when resecting a brain tumor. Nowadays, it is well known that damaging subcortical white matter tracts can bring severe neurological deficits and affect patients’ outcomes even more negatively than a cortical injury (5, 7, 35). For example, a CST interruption can bring irreversible hemiplegia while cortical damage of the motor strip may result “only” in a focal motor deficit. As it concerns language, a similar consideration can be done: in fact, the classical localist view of “well-defined” Broca’s and Wernicke’s areas has shifted to a hodotopical view (41, 43, 44). In this novel understanding of language organization, there are two major subcortical complex networks constituted by the dorsal stream which is responsible for phonological tasks and a ventral stream in charge of semantic functions (43, 45). Such language organization emphasizes the role of subcortical connections rather than the importance of definite cortical areas (44, 46–48). Moreover, it has been shown that cortical areas can undergo brain plasticity phenomena, much more relevant than white matter tracts (6, 49). Based on these considerations, it becomes clear that the possibility to know in advance the location of the main white matter tracts and their relationships with a brain tumor constitutes a fundamental help for neurosurgeons approaching a brain tumor resection (50–53). DTI allows to reconstruct and visualize the most important white matter tracts on anatomical MR images of patients, permitting to build a personalized and complete surgical planning. The tractography and the 2D reconstructions of the main fiber bundles can be uploaded on neuronavigation devices (which may be integrated with intraoperative ultrasounds) giving also real-time imaging of the white matter tracts during surgery.

Nevertheless, all the DTI surgical information has to be verified by IONM and it is nowadays unacceptable to operate close to the main fiber bundles (or eloquent cortical areas) without IONM (13, 34, 35, 45, 54). The scientific evidence supporting the use of IONM has been growing in recent years, and many studies have shown that patients’ permanent morbidity is reduced and tumor EOR is increased when IONMs are applied (12, 31, 55). Parallelly, multiple studies have demonstrated that the EOR correlates positively with OS for both LGG and HGG pushing more and more surgeons toward maximal or even supra-maximal tumor resections according to function boundaries (56–58). To date, no standardization exists regarding IONM and great variability occurs in their application to the surgical setting. Some authors use only cortical and subcortical brain mapping while others apply extensively neurophysiological techniques to have the maximal information in the operating room (31, 34, 59).

Given these premises (usefulness of DTI and need of IONM) but also the trend toward reduced invasiveness of surgical procedures, our group tried to combine IONM (fundamental), DTI (useful), and small craniotomy to verify their possibility of combination and the patients’ outcomes in a large consecutive series of gliomas. Minimally invasive neurosurgery has gained popularity thanks to several technical advancements including preoperative advanced imaging (MRI, fMRI, DTI), neuronavigation, intraoperative imaging techniques (MRI, CT, US), and microscopic and endoscopic refinements. For brain tumors, the limited invasiveness has brought to propose even day surgery plans with doubtless advantages for patients and healthcare systems (60–62). Other clear advantages of small or limited craniotomy include reduced surgical times, shorter anesthesia, and probably fewer complications (63, 64). Despite that few data are available in the neurosurgical literature regarding complications, it appears reasonable that infections, postoperative hematomas, wound problems, and consequently, neurological outcomes could be positively affected by reduced invasiveness. An indirect proof of concept stands in the observation that most, if not all, neurosurgical departments have progressively reduced the size of the craniotomy in the last 20 years with shorter operating room times (31, 60–62, 65). Parallelly, in all other surgeries (abdominal, thoracic, etc.), the rate of minimally invasive procedures is constantly growing as compared to traditional open surgery.

The first result of our series of 103 gliomas is that, in all the cases, a tumor-targeted approach resulted to be feasible without affecting the EOR or the surgical outcomes. In particular, despite most centers using IONM performing large craniotomies to allow exposure of eloquent areas to be sure of not obtaining falsely negative brain mapping, we deliberately chose to minimize the craniotomy (without reducing the IONM) considering the expertise and trust in IONM. In other words, our confidence in IONMs and our team of neurophysiologists was so high that we were confident that a negative mapping, even without observing positive responses, was a real negative. Such confidence was supported by the extensive use of DTI which, through the reconstruction of the white matter bundles’ tridimensional architecture, allowed us to tailor the craniotomy and the type of IONM, as well as the surgical corridor, for a safer and faster surgical resection. In this paper, we were also able to summarize the type of IONM according to the location of the tumor and the distance from CST (>3 cm or less than 3 cm) to make our experience reproducible by other neurosurgical centers (**Table 1**). Briefly, our technique, being relatively mini-invasive, pointed out that satisfactory motor monitoring can be achieved through TC MEPs when the lesion is not nearby the motor area. Nevertheless, in this setting DES of subcortical tracts is fundamental and should be better performed with the monopolar stimulator. On the other hand, when the tumor is near the motor area then TC MEP may not be useful and MEP should be performed with a cortical strip while mapping with monopolar and, in case, bipolar stimulation. HF monopolar stimulation demonstrated to be an efficient and reliable tool with equal safety and efficacy when compared to bipolar stimulation techniques (66, 67). Furthermore, HF monopolar stimulation showed high reliability even when CST is infiltrated by the tumor (68). HF stimulation is also related to reduced incidence of intraoperative seizures and easy transition to continuous monitoring of motor pathways during surgical resection (69). In the case of tumors located in M1, of which the macroscopic localization on the cortex is not evident, we associated a cortical bipolar LF electrical stimulation to confine the corticectomy to the minimum necessary, then switched to HF monopolar electrical stimulation when the corticectomy was completed (34). When the tumor clearly reached the cortical surface, we did not recur to LF bipolar stimulation since the cortical entry point was obviously represented by the pathological cortex and more focality of the stimulus was not needed.

Awake surgery, in our experience, was not incompatible with small craniotomies and indeed appeared to be faster and very tolerated by the patients as well. Even if the absence of positive responses may be disturbing for the surgeon, we did not recur to higher stimulation intensities or repeated stimulations to achieve positive responses, thus keeping the rates of intraoperative electric complications low. In our experience and, as already published, the use of the currents able to induce motor response was the key for having a reliable mapping.

Our experience suggests that negative mapping is safe and feasible. In this view, intraoperative DTI guidance of DES allowed us to focus the cortical and subcortical mapping to the regions of strict relationship between the tumor and the main fiber bundles and increased our trust in mapping results, even for negative mapping, since we rarely experienced major discrepancies between the two localizing techniques. Nevertheless, we always kept in mind the limitations of DTI fiber reconstruction, carefully exploring by DES the regions of uncertainty, affected by peritumoral edema or marked anatomical disruption, where DTI-FT may fail to adequately reconstruct essential bundles. In doubtful cases, resection was ultimately guided by IONM results. The neurological deficits in our series, in fact, were similar to the best available literature since only 3% of patients experienced a permanent neurological worsening (12, 70). The rate of definitive neurological deficits is related not only to preservation of white matter fiber bundles but also to safe manipulation of vascular structures. Small craniotomies and tailored surgical corridors can reduce the exposure of vessels and, consequently, undesired injury to them. This might have contributed to the low rate of definitive neurological deficits.

The EOR was not affected by the small craniotomy since the median EOR was 100% among different types of gliomas. As consequent, and again in line with the literature, OS was relatively good with a median of 15 months for GBM (or GBM-like) and as it concerns LGG, median OS was not reached after a median follow-up of 19 months (range 3–108). Kaplan–Meier survival curves are available in **Figures 8**–**11** and in supplementary materials **Figures 10A, B**, **Figures 11A, B**.

Finally, we believe that DTI and the expertise reached by our group in the use of IONM played a major role in permitting the use of small craniotomies as well as negative mapping techniques. These latter considerations may constitute a limit of our paper and may justify the reason why limited literature is available on the topic.

The effect of brain shift on preoperative DTI needs also to be discussed, even if small craniotomies are theoretically less affected by it. Since the displacement of major white matter tracts is somehow unpredictable, the authors strongly suggest checking their position during tumor resection by IONM, increasing the employment of DES mostly in the deepest part of the resection cavity, where the effects of brain shifts are likely to be amplified. Intraoperative ultrasounds and CT might be employed to correct the brain shift, as improving guidance of DES, while intraoperative MRI could even allow intraoperative DTI and fiber tracking.

As last consideration, in selected cases, the small craniotomies, as defined above, could be extended a little more to nearby non-eloquent areas, as per preoperative DTI and functional assessment, to allow more extensive resection when feasible.

## Conclusion

Reduced invasiveness (in general) and patient integrity represent surgical aims we should all try to pursue, despite technical difficulties, in the primary interest of our patients. Extensive preoperative workout, including DTI and patient cognitive assessment, allows tailoring craniotomies and IONMs to patient needs, reducing surgical invasiveness while guaranteeing optimal functional and survival outcomes. Like all surgical techniques, a learning curve is needed for relying on negative mapping as, in our experience, we found its dependability to be related to expertise, confidence, and cooperation among all the involved professionals.

Other preoperative tools have gained popularity in recent years, such as the preoperative transcranial magnetic stimulation (TMS) (71–76). As a future perspective, our team aims at evaluating and validating the preoperative TMS as a useful tool to improve reduced invasiveness and reliable negative mapping.

## Data availability statement

The original contributions presented in the study are included in the article/**Supplementary Material**. Further inquiries can be directed to the corresponding author.

## Ethics statement

Ethical review and approval were not required for the study on human participants in accordance with the local legislation and institutional requirements. Written informed consent for participation was not required for this study in accordance with the national legislation and the institutional requirements.

## Author contributions

GCa, GF, AC: concept and drafting. CB, LB: neurophysiological monitoring and data collection. BZ, GF: statistical analysis. GCo, FT: DTI reconstructions; data collections; imaging revision. AR, MC, GB: clinical records analysis and data collection. ML; CG; GCa: critical review of the manuscript. All authors contributed to the article and approved the submitted version.

## Funding

This study was supported by "Associazione Amici della Clinica Neurochirurgica" and "Università degli studi di Milano-Bicocca".

## Conflict of interest

The authors declare that the research was conducted in the absence of any commercial or financial relationships that could be construed as a potential conflict of interest.

## Publisher’s note

All claims expressed in this article are solely those of the authors and do not necessarily represent those of their affiliated organizations, or those of the publisher, the editors and the reviewers. Any product that may be evaluated in this article, or claim that may be made by its manufacturer, is not guaranteed or endorsed by the publisher.
