# Supplementary figures and images for: Diffusion tensor imaging, intra-operative neurophysiological monitoring and small craniotomy: Results in a consecutive series of 103 gliomas
Source: Front Oncol. 2022 Sep 13;12:897147. doi: 10.3389/fonc.2022.897147 (PMC9513471; doi:10.3389/fonc.2022.897147)

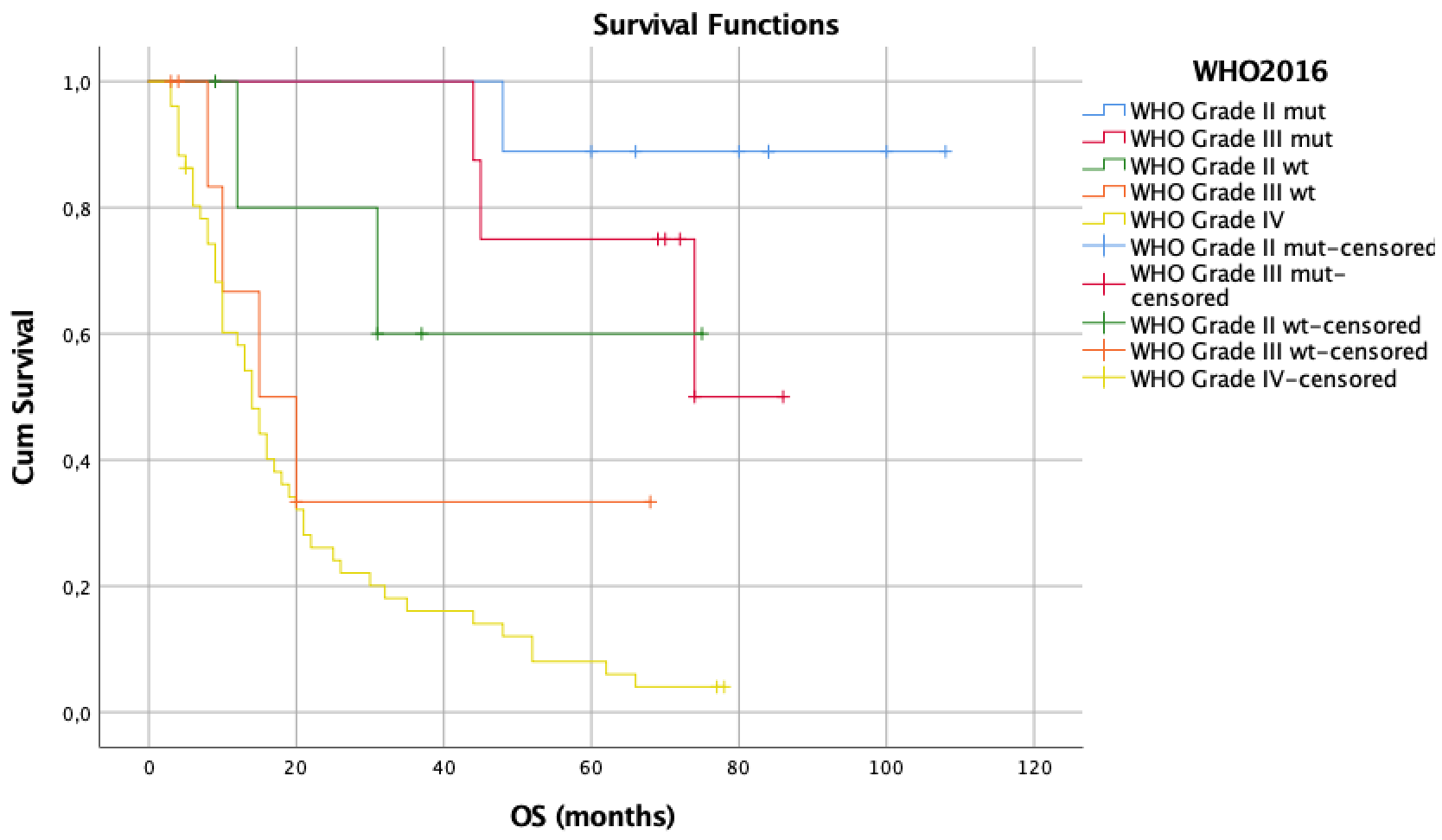

Supplement: Supplementary file 1 [file Image_1.tiff]

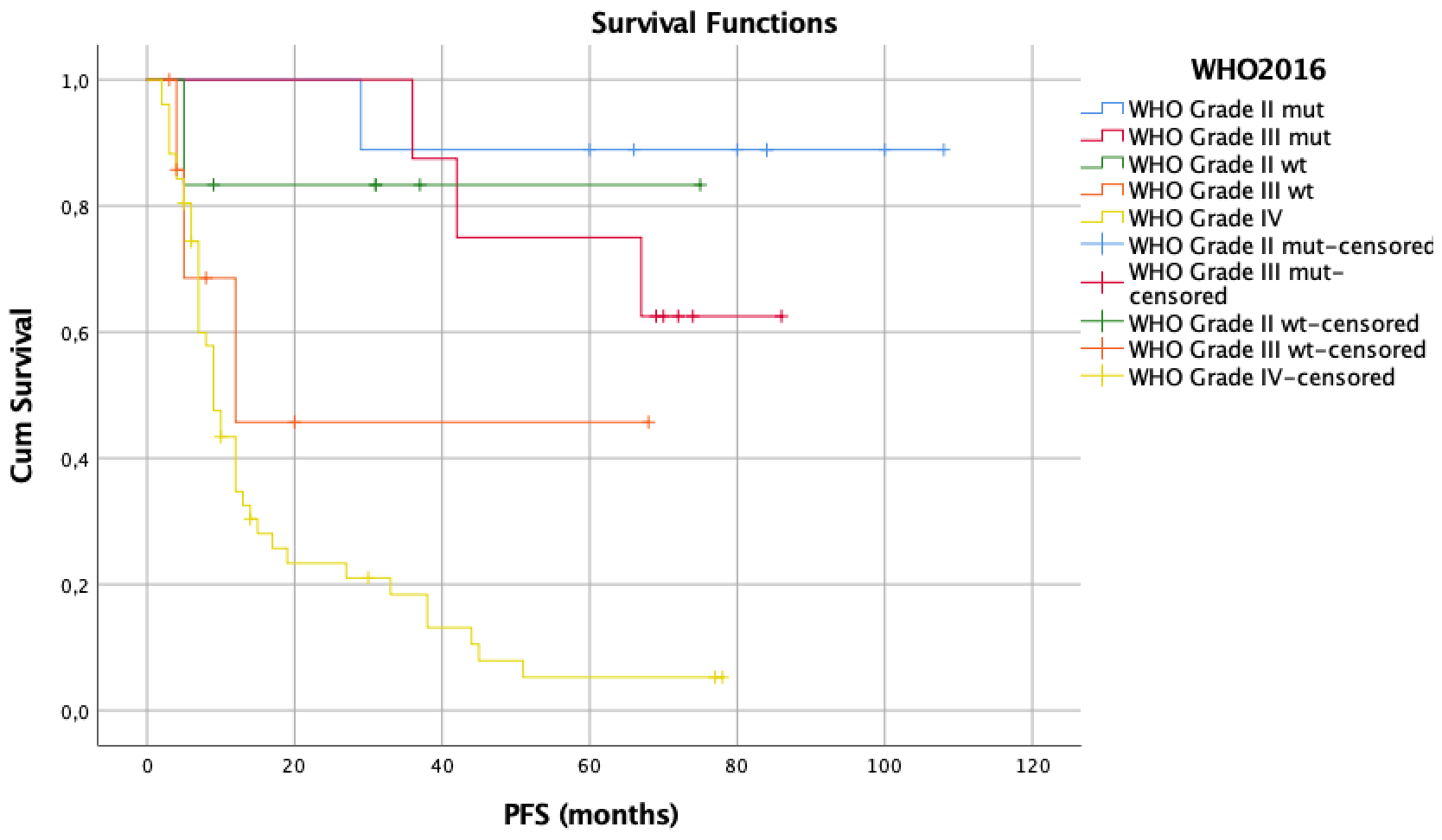

Supplement: Supplementary file 2 [file Image_2.tiff]

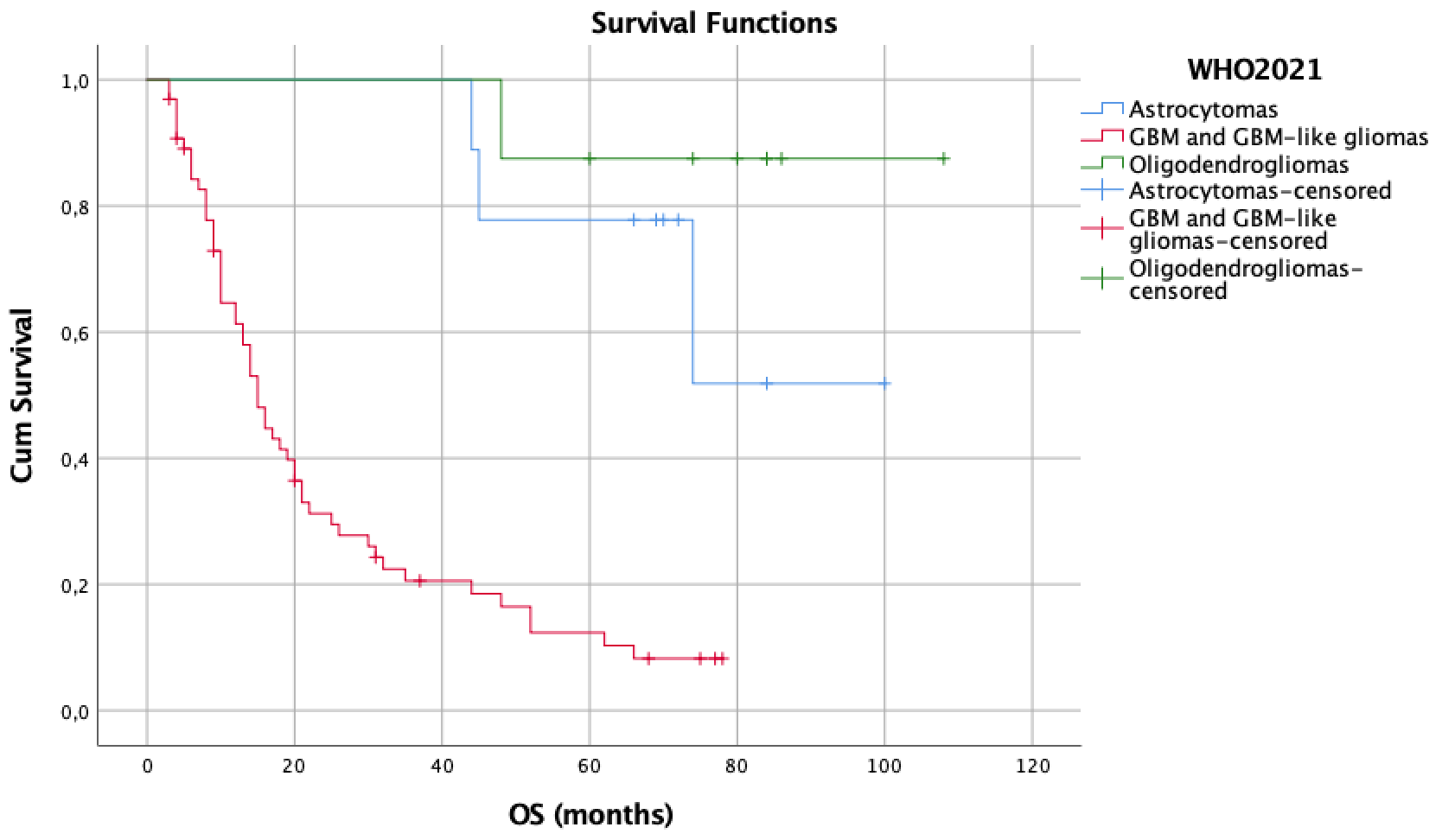

Supplement: Supplementary file 3 [file Image_3.tiff]

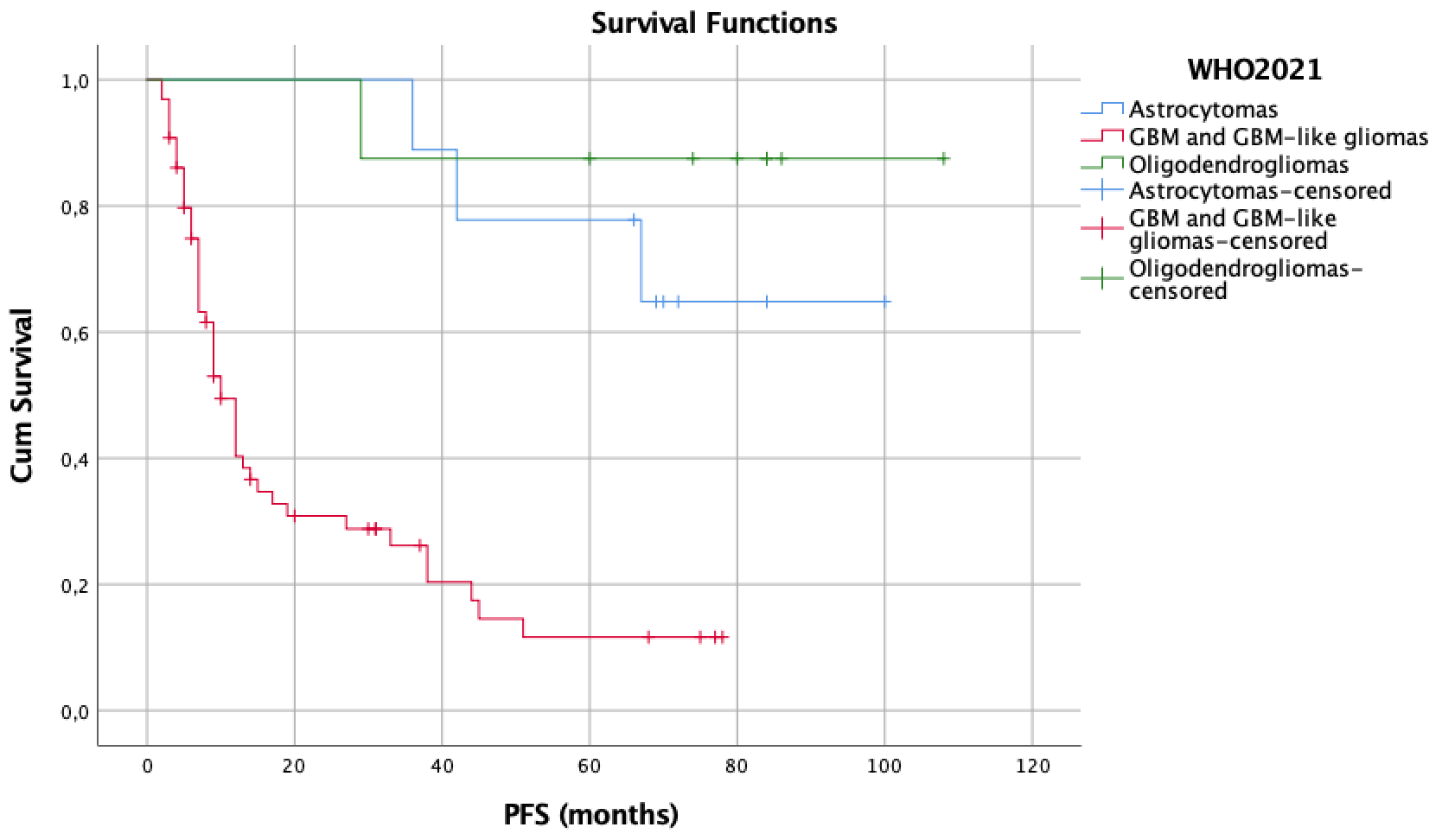

Supplement: Supplementary file 4 [file Image_4.tiff]
