# Supplementary material for: Diffusion tensor imaging, intra-operative neurophysiological monitoring and small craniotomy: Results in a consecutive series of 103 gliomas
Source: Front Oncol. 2022 Sep 13;12:897147. doi: 10.3389/fonc.2022.897147 (PMC9513471; doi:10.3389/fonc.2022.897147)
Supplement: Supplementary file 5 [file Table_1.docx]

The whole multiple linear regression model being implemented in this study is available in Supplementary Material S1 and S2

| Model Summary | | | | | |  |  |
| --- | --- | --- | --- | --- | --- | --- | --- |
| Model | R | R Square | Adjusted R Square | Std. Error of the Estimate | |  |  |
| 1 | 0.549 | 0.301 | 0.294 | 8.15319 | |  |  |
| 2 | 0.660 | 0.435 | 0.406 | 7.47888 | |  |  |
| 3 | 0.661 | 0.436 | 0.401 | 7.51042 | |  |  |
| **4** | 0.694 | **0.482** | **0.419** | 7.39698 | |  |  |
|  |  |  |  |  |  |  |  |
| Coefficients* |  |  |  |  |  |  |  |
| Model |  |  |  |  | Sig. | 95,0% Confidence Interval for B | |
|  |  | B | Std. Error | Beta |  | Lower Bound | Upper Bound |
| 1 | (Constant) | -2.694 | 1.223 |  | 0.03 | -5.12 | -0.267 |
|  | Preoperative Volume | 0.179 | 0.027 | 0.549 | 0.0001 | 0.125 | 0.233 |
| 2 | (Constant) | -3.937 | 1.228 |  | 0.002 | -6.375 | -1.499 |
|  | Preoperative Volume | 0.156 | 0.026 | 0.477 | 0.0001 | 0.105 | 0.207 |
|  | WHO Grade III IDH1/2wt | 1.899 | 2.669 | 0.056 | 0.479 | -3.399 | 7.197 |
|  | WHO Grade III IDH1/2mt | 4.16 | 2.556 | 0.128 | 0.107 | -0.914 | 9.234 |
|  | WHO Grade II IDH1/2mt | 5.052 | 2.28 | 0.174 | 0.029 | 0.526 | 9.577 |
|  | WHO Grade II IDH1/2wt | 14.319 | 3.238 | 0.347 | 0.0001 | 7.893 | 20.745 |
| 3 | (Constant) | -3.566 | 1.502 |  | 0.02 | -6.547 | -0.585 |
|  | Preoperative Volume | 0.157 | 0.026 | 0.48 | 0.0001 | 0.106 | 0.208 |
|  | WHO Grade III IDH1/2wt | 1.796 | 2.691 | 0.053 | 0.506 | -3.546 | 7.138 |
|  | WHO Grade III IDH1/2mt | 4.073 | 2.575 | 0.125 | 0.117 | -1.039 | 9.184 |
|  | WHO Grade II IDH1/2mt | 4.943 | 2.304 | 0.17 | 0.034 | 0.37 | 9.516 |
|  | WHO Grade II IDH1/2wt | 14.111 | 3.287 | 0.342 | 0.0001 | 7.586 | 20.635 |
|  | Side | -0.657 | 1.519 | -0.034 | 0.666 | -3.672 | 2.359 |
| 4 | (Constant) | -3.928 | 1.638 |  | 0.019 | -7.182 | -0.674 |
|  | **Preoperative Volume** | 0.149 | 0.027 | 0.455 | **0.0001** | 0.095 | 0.202 |
|  | WHO Grade III IDH1/2wt | 2.121 | 2.936 | 0.062 | 0.472 | -3.712 | 7.954 |
|  | WHO Grade III IDH1/2mt | 4.371 | 2.599 | 0.134 | 0.096 | -0.791 | 9.534 |
|  | **WHO Grade II IDH1/2mt** | 4.916 | 2.308 | 0.169 | **0.036** | 0.331 | 9.501 |
|  | **WHO Grade II IDH1/2wt** | 13.746 | 3.337 | 0.333 | **0.0001** | 7.117 | 20.375 |
|  | Side | -1.413 | 1.553 | -0.073 | 0.365 | -4.498 | 1.672 |
|  | T | -1.004 | 2.205 | -0.04 | 0.65 | -5.384 | 3.377 |
|  | P | 0.854 | 2.371 | 0.031 | 0.72 | -3.856 | 5.564 |
|  | O | 2.898 | 3.58 | 0.064 | 0.42 | -4.214 | 10.01 |
|  | **Insula** | 4.99 | 2.163 | 0.204 | **0.023** | 0.693 | 9.288 |
|  | Thalamic | -0.452 | 4.216 | -0.009 | 0.915 | -8.826 | 7.922 |
| *Dependent Variable: **Postoperative Volume** |  |  |  |  |  |  |  |

Table SM1

| Model Summary | | | | | |  |  |
| --- | --- | --- | --- | --- | --- | --- | --- |
| Model | R | R Square | Adjusted R Square | Std. Error of the Estimate |  |  |  |
| 1 | 0.219 | 0.048 | 0.039 | 13.42% | |  |  |
| 2 | 0.614 | 0.377 | 0.345 | 11.07% | |  |  |
| 3 | 0.619 | 0.383 | 0.345 | 11.08% | |  |  |
| **4** | 0.700 | **0.49** | **0.428** | 10.35% | |  |  |
|  |  |  |  |  |  |  |  |
| Coefficients* | |  |  |  |  |  |  |
| Model |  |  |  |  | Sig. | 95,0% Confidence Interval for B | |
|  |  | B | Std. Error | Beta |  | Lower Bound | Upper Bound |
| 1 | (Constant) | 96.035 | 2.013 |  | 0 | 92.041 | 100.028 |
|  | Preoperative Volume | -0.101 | 0.045 | -0.219 | 0.026 | -0.19 | -0.012 |
| 2 | (Constant) | 99.235 | 1.819 |  | 0 | 95.625 | 102.845 |
|  | Preoperative Volume | -0.047 | 0.038 | -0.101 | 0.221 | -0.122 | 0.029 |
|  | WHO Grade III IDH1/2wt | -3.261 | 3.953 | -0.068 | 0.411 | -11.107 | 4.584 |
|  | WHO Grade III IDH1/2mt | -14.284 | 3.786 | -0.311 | 0.0001 | -21.798 | -6.771 |
|  | WHO Grade II IDH1/2mt | -14.241 | 3.377 | -0.347 | 0.0001 | -20.943 | -7.539 |
|  | WHO Grade II IDH1/2wt | -27.083 | 4.795 | -0.466 | 0.0001 | -36.599 | -17.567 |
| 3 | (Constant) | 97.978 | 2.215 |  | 0 | 93.582 | 102.375 |
|  | Preoperative Volume | -0.05 | 0.038 | -0.109 | 0.19 | -0.126 | 0.025 |
|  | WHO Grade III IDH1/2wt | -2.912 | 3.969 | -0.06 | 0.465 | -10.79 | 4.965 |
|  | WHO Grade III IDH1/2mt | -13.987 | 3.798 | -0.304 | 0.0001 | -21.525 | -6.448 |
|  | WHO Grade II IDH1/2mt | -13.871 | 3.397 | -0.338 | 0.0001 | -20.614 | -7.127 |
|  | WHO Grade II IDH1/2wt | -26.376 | 4.847 | -0.454 | 0.0001 | -35.998 | -16.755 |
|  | Side | 2.228 | 2.24 | 0.081 | 0.322 | -2.218 | 6.675 |
| 4 | (Constant) | 99.187 | 2.292 |  | 0 | 94.634 | 103.739 |
|  | Preoperative Volume | -0.027 | 0.037 | -0.058 | 0.474 | -0.101 | 0.047 |
|  | WHO Grade III IDH1/2wt | -0.539 | 4.108 | -0.011 | 0.896 | -8.699 | 7.622 |
|  | **WHO Grade III IDH1/2mt** | -14.752 | 3.636 | -0.321 | **0.0001** | -21.974 | -7.53 |
|  | **WHO Grade II IDH1/2mt** | -13.988 | 3.229 | -0.341 | **0.0001** | -20.402 | -7.574 |
|  | **WHO Grade II IDH1/2wt** | -23.835 | 4.669 | -0.41 | **0.0001** | -33.109 | -14.561 |
|  | Side | 3.892 | 2.173 | 0.142 | 0.077 | -0.424 | 8.208 |
|  | T | 0.027 | 3.085 | 0.001 | 0.993 | -6.101 | 6.155 |
|  | P | -1.908 | 3.317 | -0.049 | 0.567 | -8.497 | 4.681 |
|  | O | -6.147 | 5.009 | -0.097 | 0.223 | -16.096 | 3.803 |
|  | **Insula** | -11.383 | 3.027 | -0.331 | **0.0001** | -17.395 | -5.37 |
|  | Thalamic | -10.504 | 5.898 | -0.149 | 0.078 | -22.219 | 1.211 |
| * Dependent Variable: **Extent of Resection** |  |  |  |  |  |  |  |

Table SM 2
